# Structural characterization of an RG-II-associated complex pectic polysaccharide from *Typha angustifolia* pollen with dendritic cell-modulating activity

**DOI:** 10.1186/s13020-026-01515-9

**Published:** 2026-09-28

**Authors:** Yongbin Xu, Mengya Guo, Yupeng Liu, Jie Chen, Songshan Shi, Chenchen Shi, Shunchun Wang, Huijun Wang

**Affiliations:** 1 The State Key Laboratory of Discovery and Utilization of Functional Components in Traditional Chinese Medicine, The MOE Key Laboratory for Standardization of Chinese Medicines, SATCM Key Laboratory for New Resources, Quality Evaluation of Chinese Medicines, Institute of Chinese Materia Medica, Shanghai University of Traditional Chinese Medicine, 1200 Cailun Road, Shanghai, 201203 PR China; 2 Longhua Hospital, Shanghai University of Traditional Chinese Medicine, Shanghai, 200032 China; 3 Shanghai University of Traditional Chinese Medicine, 1200 Cailun Road, Shanghai, 201203 China; 4 Academy of Integrative Medicine, Shanghai University of Traditional Chinese Medicine, Shanghai, 201203 China

**Keywords:** *Typha angustifolia* pollen, Pectic polysaccharide, Dendritic cell maturation, Cytotoxic T-cells activation, Structure–activity relationship

## Abstract

**Background:**

Pectic polysaccharides from medicinal plants are promising natural immunomodulators, but their structural heterogeneity has hindered the identification of bioactive domains and limited their further development. The structural basis underlying their regulation of dendritic cell-mediated immune responses remains poorly understood.

**Purpose:**

This study aimed to characterize PTPS-1-3, a pectic polysaccharide isolated from *Typha angustifolia* pollen, and evaluate its effects on dendritic cell maturation and antigen-specific T-cell activation.

**Methods:**

PTPS-1-3 was characterized by molecular weight determination, monosaccharide composition analysis, methylation analysis, multidimensional nuclear magnetic resonance spectroscopy, and partial acid hydrolysis. Its immunomodulatory activity was evaluated in bone marrow-derived dendritic cells (BMDCs) by assessing cell viability, maturation markers, cytokine secretion, and phagocytic capacity. Transcriptomic analysis and an OT-I co-culture system were used to investigate the underlying pathways and antigen-specific CD8⁺ T-cell responses. Structure–activity relationships were explored by comparison with a partial acid hydrolysate and a homogalacturonan-type polysaccharide.

**Results:**

PTPS-1-3 was an approximately 10 kDa pectic polysaccharide composed mainly of rhamnose, arabinose, xylose, galactose, and galacturonic acid. It contained homogalacturonan regions, arabinogalactan-II-rich side chains, and rhamnogalacturonan-II-associated motifs. PTPS-1-3 promoted BMDC maturation without detectable cytotoxicity, as shown by increased CD40, CD80, and CD86 expression, enhanced TNF-α, IL-1β, and IL-6 secretion, and reduced phagocytic capacity. Transcriptomic analysis revealed enrichment of antigen processing and presentation pathways. PTPS-1-3-treated BMDCs further enhanced antigen-specific CD8⁺ T-cell activation, proliferation, and IFN-γ and TNF-α production. Comparative analysis suggested that the immunomodulatory activity of PTPS-1-3 may be associated with its intact and complex branched pectic architecture, although differences in molecular weight may also contribute to the observed activity.

**Conclusion:**

PTPS-1-3 promotes BMDC maturation and enhances subsequent antigen-specific CD8⁺ T-cell responses. Its immunomodulatory activity may be associated with the integrity and complexity of its pectic architecture, while the contribution of molecular weight and individual structural features requires further clarification.

**Graphical abstract:**

**Figure:**
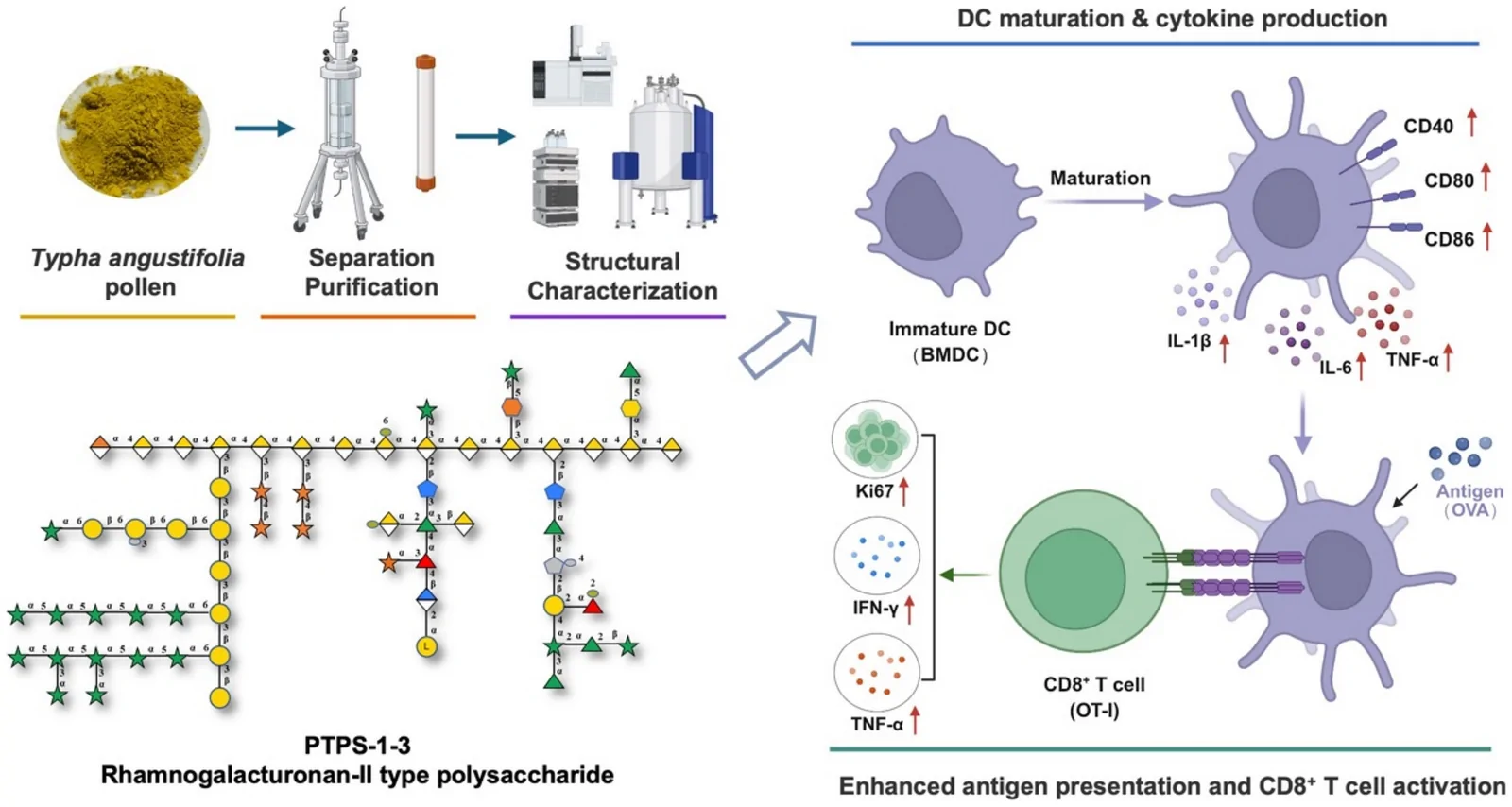

**Supplementary Information:**

The online version contains supplementary material available at https://doi.org/10.1186/s13020-026-01515-9.

## Introduction

Medicinal resource-derived polysaccharides are increasingly recognized for their multiple biological functions and good biocompatibility, making them a focus of intensive research [1]. However, unlike structurally defined small molecules, plant polysaccharides possess highly heterogeneous, hierarchically organized architectures [2]. Their biological functions are intimately governed by multiple structural parameters, including chain size, monosaccharide types, linkage types, branching architecture, and the presence of uronic acid-rich domains. This inherent heterogeneity significantly impedes the detailed elucidation of structure and the interpretation of biological function [3]. Among polysaccharides, pectin stands out for its unique structural complexity, with a molecular backbone composed of smooth regions of homogalacturonan (HG) and hairy regions rich in rhamnogalacturonan-I (RG-I) and RG-II. Although classical analytical approaches such as methylation analysis and NMR spectroscopy have been widely used, accurate domain-level assignment remains challenging due to overlapping signals, low-abundance RG-II-associated motifs, and structural heterogeneity. In particular, AG-II-rich side chains and RG-II-related structures are difficult to resolve within intact pectic matrices, limiting the understanding of their domain-specific organization.

Accumulating evidence suggests that pectic polysaccharides can modulate immune responses through interactions with innate immune cells, including dendritic cells [4–6]. As specialized antigen-presenting cells, DCs are essential for linking innate immune recognition to adaptive immune responses. Immature DCs are characterized by strong antigen uptake capacity but relatively low expression of co-stimulatory molecules, whereas mature DCs display enhanced antigen-presenting ability, increased secretion of cytokines and up-regulated expression of surface molecules such as CD40, CD80 and CD86 [7, 8]. Such alterations in phenotype and function are essential for launching adaptive immunity. Among adaptive immune effectors, CD8⁺ T cells play a central role in antitumor immunity by recognizing tumor-associated antigens presented by major histocompatibility complex class I molecules and directly eliminating malignant cells. Upon activation, cytotoxic CD8⁺ T cells exert their effector functions through the production of cytokines such as IFN-γ and TNF-α, which contribute to tumor cell killing and immune-mediated tumor control [9, 10]. A growing body of evidence indicates that natural polysaccharides can stimulate dendritic cell maturation, likely by engaging pattern-recognition receptors and subsequently activating downstream immune signaling cascades [11, 12]. Therefore, bone marrow-derived dendritic cells (BMDCs) provide a useful cellular model for evaluating the immune-supporting potential of structurally defined food-derived polysaccharides.

*Typha angustifolia* L. pollen is a well-known ethnopharmacological agent in East Asia, where it has been used traditionally for centuries. It has been associated with multiple biological activities, including hemostatic, anti-inflammatory, immunoregulation and circulation-promoting effects [13]. The main component responsible for the immune regulatory effect is its polysaccharide content [14]. In our previous work, a pectic polysaccharide fraction, designated PTPS-1-3, was isolated from *Typha angustifolia* pollen and found to be rich in arabinose and galactose, with structural features suggestive of AG-II-rich regions and RG-II-related domains [15]. Although PTPS-1-3 has been previously isolated and its preliminary structural characteristics and RG-II content have been reported, its detailed structural organization and immunomodulatory properties remain largely unexplored. Therefore, the present study aimed to comprehensively characterize the fine structure of PTPS-1-3 using methylation analysis and multidimensional NMR spectroscopy, with particular emphasis on its complex pectic domains. Furthermore, its effects on BMDC activation, maturation, and antigen-specific CD8⁺ T-cell responses were systematically evaluated to establish a preliminary understanding of its structure–activity relationship.

## Materials and methods

### Isolation and purification of PTPS-1-3

**Fig. 1:**
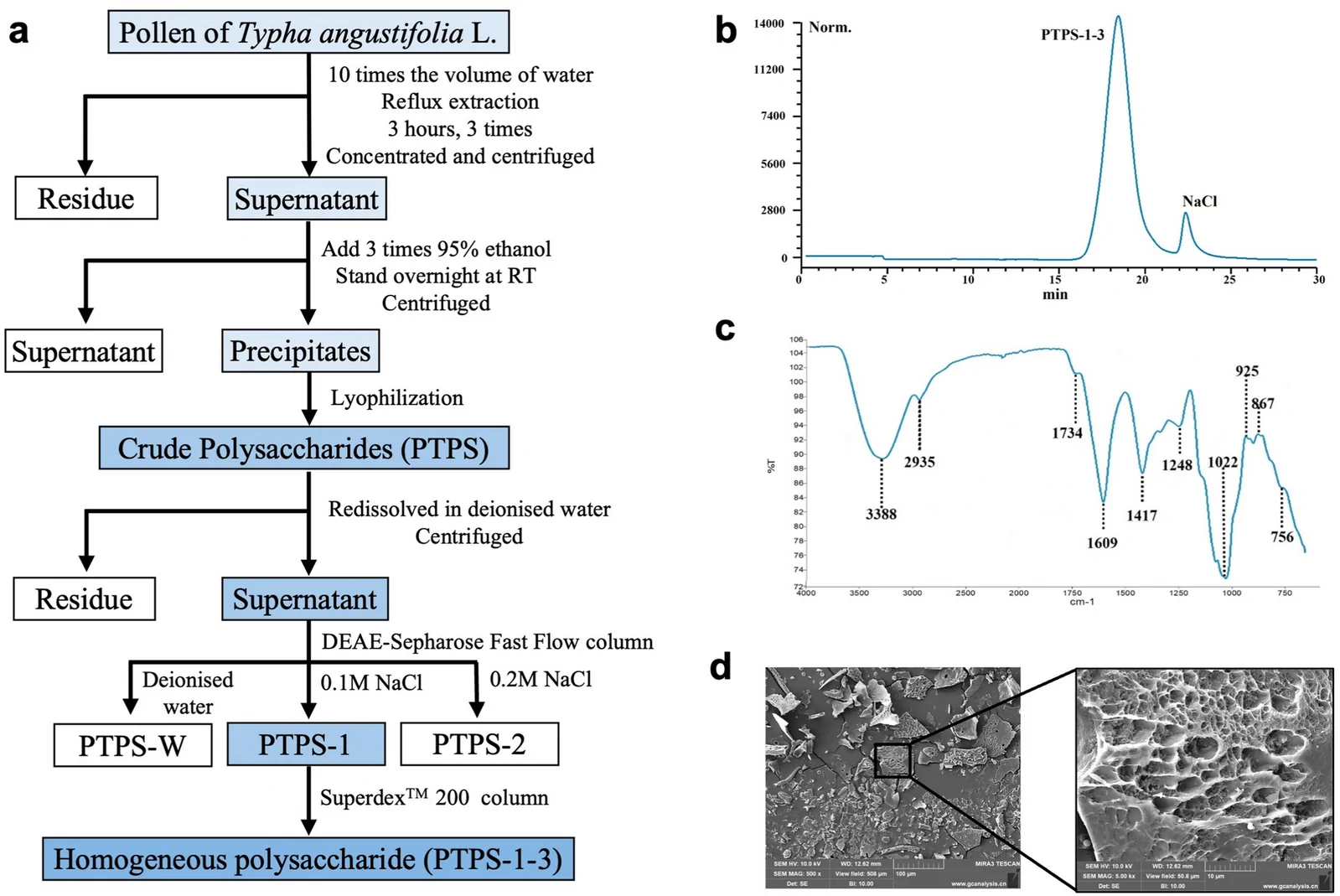
**a** Purification scheme of PTPS-1-3 from *Typha angustifolia* pollen. **b** Molecular weight distribution of PTPS-1-3 determined by HPGPC [15]. **c** FT-IR spectrum of PTPS-1-3. **d** SEM images of PTPS-1-3 at 500× (left) and 5000× (right)

The pollen of *Typha angustifolia* was purchased from Shanghai Kangqiao Pharmacy (Shanghai, China). The material was authenticated as the pollen of *Typha angustifolia* L. The sample used in this study was obtained from a single batch. Supplementary materials provide further details on the remaining reagents and materials. PTPS-1-3 was extracted from *Typha angustifolia* pollen according to our previously established protocol [15], and the overall workflow is summarized in Fig. 1a.

### FT-IR analysis

FT-IR spectra of PTPS-1-3 were recorded using a PerkinElmer Spectrum Two spectrometer. Samples (2 mg) were scanned over the range of 4000–400 cm⁻^1^ for structural feature identification.

### SEM analysis

Surface morphology was examined by scanning electron microscopy. Dried PTPS-1-3 powder was affixed to conductive adhesive stubs and sputter-coated with gold using an MC1000 ion coater (Hitachi, Japan). Micrographs were subsequently recorded at 3.0 kV under high vacuum on an SU8100 scanning electron microscope (Hitachi, Japan) at various magnifications.

### Methylation analysis

Methylation analysis was conducted according to a previously described protocol [16] with minor modifications. Briefly, polysaccharide samples (10 mg) were dried over P_2_O_5_ under vacuum for 2 days and dissolved in 2 mL anhydrous DMSO under a nitrogen atmosphere. Freshly ground dry NaOH powder (200 mg) was added, and the mixture was stirred for 1 h. Methylation was performed by dropwise addition of CH_3_I (1.5 mL) under nitrogen protection and light avoidance, followed by continuous stirring at room temperature for 2 h. The reaction was terminated by adding distilled water, and the methylated polysaccharides were recovered by dialysis and lyophilization. The methylation procedure was repeated 3–5 times until complete methylation was achieved, as confirmed by GC–MS analysis.

Fully methylated polysaccharides were hydrolyzed, reduced, and acetylated to generate partially methylated alditol acetates (PMAAs) according to the previously described procedure. The resulting PMAA derivatives were analyzed by GC–MS (Agilent 7890B-5977A) equipped with an HP-5MS capillary column (60 m × 0.25 mm × 0.25 μm). Helium was used as the carrier gas at a flow rate of 1 mL/min. The oven temperature program was set as follows: initial temperature 140 °C, increased to 180 °C at 2 °C/min, then to 200 °C at 1 °C/min, and finally to 250 °C at 3 °C/min and maintained for 5 min. MS detection was performed using an electron ionization (EI) source at 70 eV, with an ion source temperature of 230 °C, quadrupole temperature of 150 °C, and scanning range of m/z 40–500. PMAAs were identified based on their retention times and characteristic mass fragmentation patterns by comparison with reported standards and previously published assignments. The relative molar proportions of glycosyl linkages were calculated from the peak areas of individual PMAA derivatives after normalization, and the linkage composition was expressed as molar percentages.

### NMR analysis

For deuterium exchange, 60 mg of PTPS-1-3 was dissolved in 1 m L D_2_O (99.8% D) and lyophilized. This procedure was repeated two times to sufficiently replace exchangeable protons. The deuterium-exchanged sample was finally dissolved in 0.5 mL D_2_O, giving a sample concentration of approximately 120 mg/mL, and transferred into a 5 mm NMR tube. NMR spectra were acquired at 25 °C on a Bruker Avance III 600 MHz spectrometer equipped with a PABBO probe (5 mm, BB/19F-1H/D, Z-GRD). One-dimensional ^1^H, ^13^C, and DEPT135 spectra and two-dimensional ^1^H–^1^H COSY, ^1^H–^13^C HSQC, and ^1^H–^13^C HMBC spectra were recorded using standard Bruker pulse sequences. For the NMR pulse sequences and key acquisition and processing parameters, please refer to Additional file 1: Table S1 in the Supplementary materials. Chemical shifts were referenced to internal acetone at δH 2.225 ppm and δC 31.45 ppm [17]. Raw NMR data were initially processed using Bruker TopSpin, including Fourier transformation, phase correction where applicable, baseline correction, and spectral calibration. Two-dimensional spectra were further optimized and analysed using MestReNova (version: 12.0.0-20080), including contour adjustment, peak picking, and annotation of diagnostic cross-peaks.

### BMDC generation

BMDCs were obtained from the femurs and tibiae of 8-week-old C57BL/6 mice using a standard protocol [18]. After euthanasia, bones were excised under sterile conditions, and bone marrow was flushed using cold RPMI 1640 medium. Cell suspensions were filtered and centrifuged, followed by erythrocyte lysis. BMDCs were placed in RPMI 1640 complete medium supplemented with 20 ng/mL GM‑CSF and 10 ng/mL IL‑4, and maintained under standard conditions (37 °C, 5% CO_2_). Half‑volume medium replacement with fresh cytokine‑containing medium was performed on days 3 and 5. On day 6, immature BMDCs were harvested by collecting the loosely attached and floating cells. All animal experiments were carried out in accordance with a protocol approved by the Institutional Animal Care and Use Committee of Shanghai University of Traditional Chinese Medicine (Ethics Number: PZSHUTCM2502240002).

### Cell viability

BMDCs were seeded at 2 × 10^4^ cells per well in 96-well plates. Following a 12 h pre-incubation, they were exposed to 1 μg/mL LPS or 5–40 μg/mL PTPS-1-3 for 24 h, after which metabolic activity was assessed via a commercial cell viability assay as recommended by the supplier [19].

### qRT-PCR

BMDCs were seeded, allowed to adhere, and subsequently challenged with LPS or PTPS-1-3 for 12 h. TRIzol-based RNA extraction was followed by reverse transcription, and target gene expression was measured by quantitative PCR using SYBR Green, employing primers detailed in Additional file 1: Table S5.

### ELISA

Following treatment, BMDC culture media were collected and the secretion of IL-6, IL-1β, and TNF-α was assessed by commercial ELISA kits as per the protocols provided.

### Flow cytometry

BMDCs were treated with LPS, PTPS-1-3, PTPS-1-3-on (the partial acid hydrolysate of PTPS-1-3), or FPLP (a previously characterized HG-type polysaccharide from *Ficus pumila* L. fruit [29]) for 24 h. Cells were then harvested, washed, and stained with fluorochrome-conjugated antibodies against CD11c, MHC II, CD80, CD86, and CD40, together with viability dye. For phagocytosis assays, FITC-dextran uptake was performed under standard conditions. Data acquisition was conducted using a FACSCelesta cytometer and analyzed with Fojo software.

### RNA-sequencing and transcriptomic analysis

Total RNA was extracted from BMDCs using TRIzol reagent according to the manufacturer's instructions. RNA purity and quantity were evaluated using a NanoDrop 2000 spectrophotometer, and RNA integrity was assessed using an Agilent 2100 Bioanalyzer. Three biological replicates were prepared for each group. RNA libraries were constructed using the VAHTS Universal V10 RNA-seq Library Prep Kit and sequenced on an Illumina NovaSeq X Plus platform with 150 bp paired-end reads by OE Biotech Co., Ltd. (Shanghai, China). Raw sequencing reads were processed using fastp to remove low-quality reads, and clean reads were aligned to the reference genome using HISAT2. Gene expression levels were quantified based on read counts generated by HTSeq-count. Differentially expressed genes (DEGs) were identified using DESeq2 with q value < 0.05 and fold change > 2 or < 0.5 as the cutoff criteria. Functional enrichment analyses, including GO, KEGG pathway enrichment, and gene set enrichment analysis (GSEA), were performed to identify biological pathways associated with PTPS-1-3 treatment.

### Antigen presentation assay using OT-I CD8⁺ T cells

BMDCs were differentiated from mouse bone marrow cells for 6 days, and immature BMDCs were collected for antigen presentation assays. Cells were seeded into 24-well plates at a density of 2 × 10^5^ cells/mL and stimulated for 24 h with medium control, PTPS-1-3 (40 μg/mL), ovalbumin (OVA, 100 μg/mL), or a combination of PTPS-1-3 (40 μg/mL) and OVA (100 μg/mL). After stimulation and antigen loading, BMDCs were washed with PBS to remove unbound PTPS-1-3 and OVA [20].

CD8α⁺ T cells were isolated from the spleens of OT-I mice using CD8a (Ly-2) MicroBeads (Miltenyi Biotec, Germany). Prior to co-culture, stimulated BMDCs were treated with mitomycin C (50 μg/mL) for 30 min to prevent proliferation, followed by washing with PBS and resuspension. BMDCs (2 × 10^4^ cells) were then co-cultured with OT-I CD8⁺ T cells (2 × 10^5^ cells) at a ratio of 1:10 in 96-well round-bottom plates for 48 h. GolgiStop™ was added during the final 4–6 h of co-culture.

After co-culture, cells were collected and stained for viability and surface markers, followed by intracellular staining. Dead cells were excluded using eFluor™ 780 viability dye. Cells were stained with anti-CD8α and anti-CD44 antibodies for surface markers, and anti-IFN-γ, anti-TNF-α, and anti-Ki67 antibodies after fixation and permeabilization. Flow cytometry analysis was performed, and at least 10,000 viable CD8⁺ T cells were collected for each sample.

### Statistical analysis

All data, displayed as mean ± SD of three or more independent experiments, were analyzed for statistical significance via one-way ANOVA in GraphPad Prism 10.

## Results

### Isolation and purification of PTPS-1-3

PTPS-1-3 was separated and purified from *Typha angustifolia* pollen according to a previously established purification protocol, as illustrated in Fig. 1a [15]. The chromatographic purification curve of PTPS-1-3 is shown in Additional file 1: Fig. S1. As shown in Fig. 1b, PTPS-1-3 exhibited a single, symmetric peak, corresponding to a molecular weight of approximately 10 kDa. Earlier colorimetric analysis based on thiobarbituric acid reaction had suggested the presence of RG-II-associated structural signatures[15], implying a potentially complex pectic architecture. On this basis, further structural investigations were undertaken to resolve its domain organization and fine structural features.

### FT-IR analysis

The functional groups of PTPS-1-3 were probed by FT-IR spectroscopy (Fig. 1c). A broad O–H stretching absorption was observed at 3388 cm⁻^1^. The peaks at 1609 and 1417 cm⁻^1^ corresponded to carboxylate asymmetric stretching and C–O stretching, respectively. The strong band at 1022 cm⁻^1^ reflected glycosidic C–O–C/C–O vibrations. A peak at 1734 cm⁻^1^ indicated esterified/protonated carboxyl groups, consistent with the presence of uronic acid-containing pectic domains. Collectively, these spectral features confirm the acidic nature and pectic characteristics of the polysaccharide fraction.

### SEM analysis

The microstructure of PTPS-1-3 was examined by scanning electron microscopy. At low magnification, PTPS-1-3 appeared as irregular sheet-like and fragmented aggregates with uneven particle sizes (Fig. 1d). At higher magnification, the surface showed a loose and porous architecture with rough pore walls and locally stacked lamellar textures. This irregular and porous morphology is consistent with the amorphous aggregation of polysaccharide chains after drying and may reflect its highly branched and heterogeneous molecular architecture.

### Monosaccharide composition analysis

PTPS-1-3 exhibited a uronic acid content of roughly 19.2%, supporting its classification as an acidic pectic polysaccharide. To further probe uronic acid-associated linkages, a reduced derivative (PTPS-1-3-Re) was prepared, while a partial acid hydrolysate (PTPS-1-3-on) was generated in parallel to enrich acid-resistant structural domains. Monosaccharide analysis indicated that the polysaccharide was mainly composed of Rha, Ara, Xyl, Gal, and GalA (Additional file 1: Fig. S2, Table S2), consistent with a typical pectic framework containing both neutral side chains and uronic acid-rich backbones. The relatively high proportions of Ara and Gal suggested the presence of arabinogalactan-type domains, whereas Rha and GalA were indicative of rhamnogalacturonan backbone structures. The detection of Xyl, together with prior thiobarbituric acid-positive results, further supported the possible presence of RG-II-related structural motifs[15]. Based on these observations, more detailed structural elucidation was subsequently carried out using methylation analysis and multidimensional NMR spectroscopy to resolve linkage patterns and domain organization.

### Methylation analysis

**Fig. 2:**
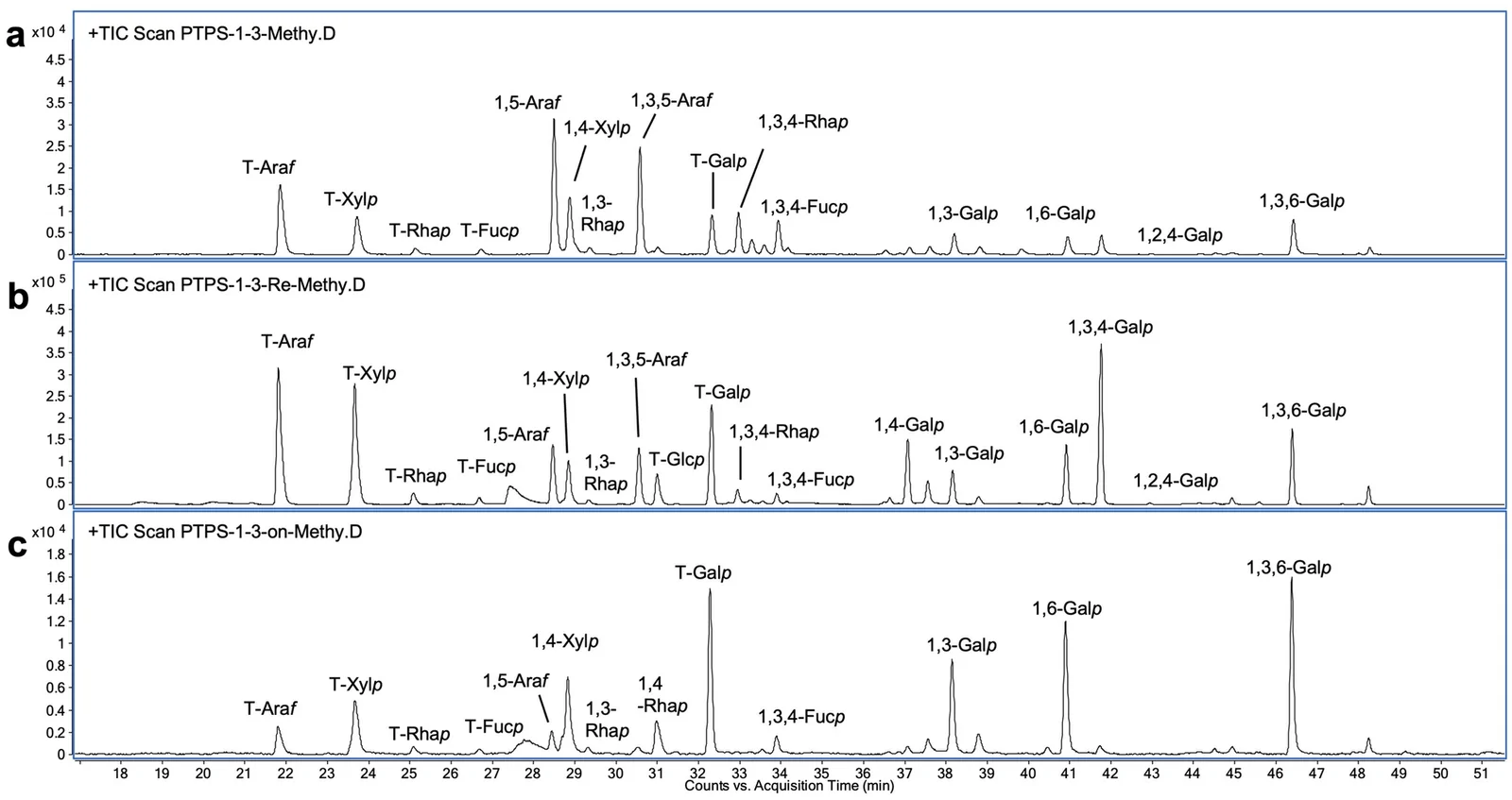
Gas chromatographic profiles of methylation analysis. The chromatograms shown are for PTPS-1-3 **a**, PTPS-1-3-Re **b**, and PTPS-1-3-on **c**. Linkage assignments were deduced from the mass spectra of the corresponding PMAA fragments

Methylation analysis was conducted for PTPS-1-3, PTPS-1-3-Re and PTPS-1-3-on to elucidate their glycosidic linkage patterns. The GC–MS profiles of the corresponding partially methylated alditol acetates are shown in Fig. 2, and the detailed linkage compositions are summarized in Additional file 1: Table S3 and Fig. S3. A total of 19 PMAA derivatives were identified in PTPS-1-3, indicating a highly branched and structurally heterogeneous polysaccharide. The major linkages included T-Ara*f*, 1,5-Ara*f*, 1,3,5-Ara*f*, T-Gal*p*, 1,3-Gal*p*, 1,6-Gal*p* and 1,3,6- Gal*p*. These linkage patterns are consistent with the presence of arabinan and arabinogalactan-type side chains. In particular, the occurrence of 1,3-Gal*p* and 1,3,6-Gal*p*, together with T- and 1,5-Ara*f*, supports the existence of AG-II-rich neutral side-chain regions in PTPS-1-3. After reduction of uronic acid residues, PTPS-1-3-Re showed the appearance of 1,4-Gal*p* and 1,3,4-Gal*p*, with relative abundances of 10.7% and 10.6%, respectively. These residues are likely derived from reduced 1,4-Gal*p*A and 1,3,4-Gal*p*A, supporting the presence of HG segments and substituted GalA-containing regions. Compared with PTPS-1-3, the partial acid hydrolysate PTPS-1-3-on showed markedly reduced proportions of T- Ara*f*, 1,5- Ara*f* and 1,3,5- Ara*f*, accompanied by an increased proportion of terminal Gal*p*. These changes suggest that arabinofuranosyl residues are mainly located in acid-labile side-chain regions attached to galactan-rich structures. Notably, several rare monosaccharide residues characteristic of RG-II domains are usually present at low abundance and are difficult to fully identify by GC–MS-based methylation analysis alone[21]. Therefore, although the methylation results support the presence of AG-II-rich side chains, HG segments and substituted GalA-containing regions, complete assignment of RG-II-related structural features requires further confirmation by multidimensional NMR spectroscopy.

### NMR analysis

**Fig. 3.**
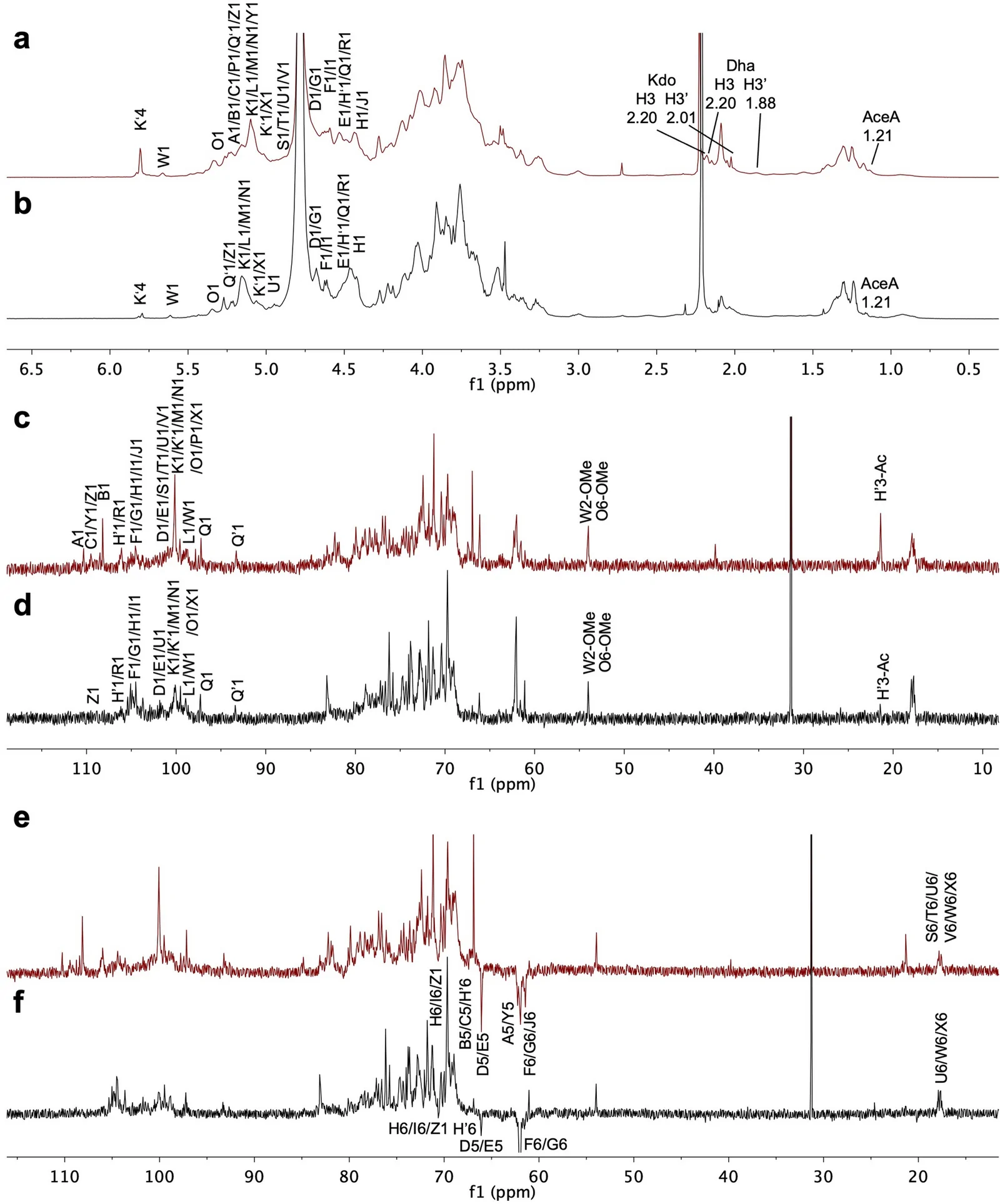
^1^H NMR spectrum of PTPS-1-3 (**a**) and PTPS-1-3-on (**b**). ^13^C NMR spectrum of PTPS-1-3 (**c**) and PTPS-1-3-on (**d**). DEPT135 NMR spectrum of PTPS-1-3 (**e**) and PTPS-1-3-on (**f**). (A. T-α-Ara*f*; B. 1.5-α-Ara*f*; C. 1,3,5-α-Ara*f*; D. T-β-Xyl*p*; E. 1,4-β-Xyl*p*; F.T-β-Gal*p*; G. 1,3-β-Gal*p*; H. 1,6-β-Gal*p*; H’. 1,6-β-Gal*p*-3-Ac; I. 1,3,6-β-Gal*p*; J. 1,2,4-β-Gal*p*; K. T-α-Gal*p*A; L. 1,4-α-Gal*p*A; M. 1,3,4-α-Gal*p*A; N. 1,2,4-α-Gal*p*A; O. 1,4-α-Gal*p*A-6-OMe; P. 1,2,3,4-α-Gal*p*A; Q. 1,4-β-Gal*p*A(red); Q’. 1,4-α-Gal*p*A(red); R. 1,2-β-Glc*p*A; S. T-α-Rha*p*; T. 1,2-α-Rha*p*; U. 1,3-α-Rha*p*; V. 1,2,3,4-α-Rha*p*; W. T-α-Fuc*p*-2-OMe; X. 1,3,4-α-Fuc*p*; Y. 1,2,3-α-Ara*p*; Z. 1,3-β-Api*f*)

**Fig. 4:**
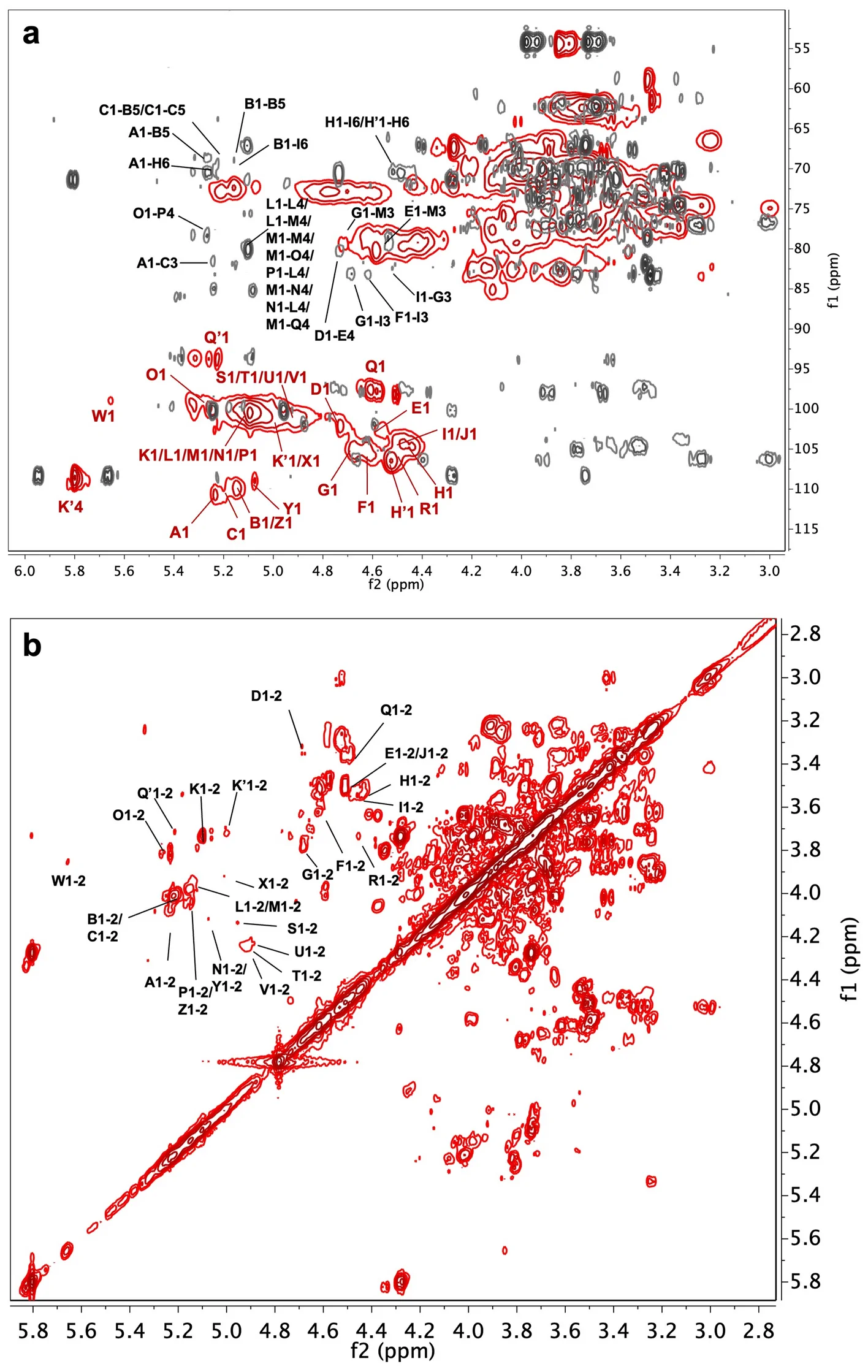
**a** Two-dimensional NMR spectra of PTPS-1-3 showing the overlay of HSQC (red) and HMBC (black) signals. Only representative cross-peaks are labeled to reduce spectral congestion. **b** ^1^H-^1^H COSY spectrum of PTPS-1-3. (The letter annotations in this figure are consistent with those in Fig. 3.)

PTPS-1-3 was further structurally characterized by NMR with ^1^H (Fig. 3a), ^13^C (Fig. 3c), HSQC and HMBC (Fig. 4a), ^1^H–^1^H COSY (Fig. 4b). To assist structural assignment and distinguish acid-labile side-chain regions from relatively stable structural segments, the partial acid hydrolysate PTPS-1-3-on was also analyzed by ^1^H and ^13^C NMR spectroscopy (Fig. 3b, d, e). Interpretation of the NMR spectra drew on monosaccharide composition, uronic acid reduction, partial acid hydrolysis, and methylation analysis, alongside known pectic polysaccharide spectral features [22, 23].

In the ^13^C NMR spectrum of PTPS-1-3, multiple anomeric carbon signals were observed in the region of 95–110 ppm, indicating the presence of diverse glycosyl residues. The relatively downfield anomeric signals at 108–110 ppm were characteristic of arabinose, whereas signals at 103–106 ppm corresponded to galactose. Specifically, the signal at 105.2 ppm was assigned to T-β-Gal*p*, while those at 104.9 and 104.6 ppm were ascribed to 1,3-β-Gal*p* and overlapping signals of 1,3,6-β-Gal*p*/1,6-β-Gal*p*, respectively. Ara*f* residues exhibited distinctly lower-field anomeric carbon signals, with 110.4 ppm assigned to terminal α-L-Ara*f*, 108.6 ppm to 1,5-α-L-Ara*f*, and 109.1 ppm to 1,3,5-α-L-Ara*f* [24]. Corresponding anomeric proton signals were confirmed in the HSQC spectrum (Fig. 4a): 4.62 ppm(T-β-Gal*p*), 4.68 ppm (1,3-β-Gal*p*), 4.49 and 4.45 ppm (1,3,6-β-Gal*p* and 1,6-β-Gal*p*, respectively), 5.24 ppm (T-α-Ara*f*), and 5.17–5.19 ppm (1,5-α-Ara*f* and 1,3,5-α-Ara*f*). The coexistence of abundant β-Gal*p* backbones substituted with α-Araf side chains is a diagnostic feature of AG-II, indicating that PTPS-1-3 contains a typical AG-II domain.

Comparison with PTPS-1-3-on further supported this assignment. After partial acid hydrolysis, the signals corresponding to Ara residues were markedly weakened in PTPS-1-3-on, especially those assigned to terminal α-Ara*f*, 1,5-α-Ara*f* and 1,3,5-α-Ara*f* (Additional file 1: Fig. S4). In contrast, galactopyranosyl-related signals were relatively retained or became more prominent. This spectral change is consistent with the methylation results, which showed decreased proportions of terminal Araf, 1,5-linked Ara*f* and 1,3,5-linked Ara*f* in PTPS-1-3-on. These results indicate that the Ara*f* residues are mainly located in acid-labile side chains attached to galactan-rich regions, further confirming the AG-II-like structural characteristics of PTPS-1-3.

Besides signals from neutral sugars, the NMR spectra clearly displayed resonances characteristic of GalpA. Anomeric signals at δH/δC 5.09/100.4 and 5.26/99.9 ppm were assigned to non-esterified and esterified 1,4-α-D-GalpA units, respectively [25], while their corresponding C-6 resonances appeared at 176.2 and 172.4 ppm [26]. These observations confirmed the presence of HG domain in PTPS-1-3. Chemical composition and methylation analyses suggested that PTPS-1-3 also contains RG-II domain, one of the most structurally complex and highly conserved domains in pectin. Although some RG-II-specific signals were weak or partially overlapped due to their relatively low abundance, several diagnostic NMR features supported the presence of this domain. In the ^1^H NMR spectrum (Fig. 3a), signals at 2.01 and 2.20 ppm were assigned to H-3 and H-3′ of 3-deoxy-D-manno-octulosonic acid (Kdo), while signals at 1.88 and 2.20 ppm corresponded to H-3 and H-3′ of 3-deoxy-D-lyxo-heptulosaric acid (Dha), both of which are hallmark components of RG-II side chains and are difficult to detect by monosaccharide composition analysis alone [21].

In addition, methyl group signals at 17.6/1.18, 17.9/1.22, 18.1/1.25, and 18.6/1.26 ppm were attributed to the C-6/H-6 resonances of α-L-Rha*p* residues in different substitution patterns, including T-α-L-Rha*p*, 1,2-α-L-Rha*p*, 1,3-α-L-Rha*p*, and 1,2,3,4-α-L-Rha*p* [27]. Additionally, the signal at 16.6/1.22 ppm was assigned to 1,3,4-α-L-Fuc*p*. Furthermore, the identification of rare sugar residues such as 1,3-β-D-apiofuranosyl (Api*f*), together with substituted Gal*p*A backbones, is consistent with the highly conserved RG-II architecture reported across plant species, in which complex side chains are attached to *O*-2 of Gal*p*A residues via β-Api*f* linkages. Based on comprehensive analysis of the NMR spectra, proton and carbon signals were assigned to 29 distinct glycosyl residues in PTPS-1-3, including various Ara*f*, Gal*p*, Xyl*p*, Gal*p*A, Rha*p*, Fuc*p*, Ara*p*, Glc*p*A, and Api*f* residues. The major ^13^C and ^1^H NMR chemical shifts of PTPS-1-3 were listed in Table 1**.**

**Table 1:** ^13^C NMR and ^1^H NMR chemical shifts (ppm) for PTPS-1-3

Residues	C-1/H-1	C-2/H-2	C-3/H-3	C-4/H-4	C-5/H-5	C-6/H-6
T-α-Ara*f*	110.4	82.4	78.4	85.0	62.3	–
(A)	5.24	4.21	4.01	4.12	3.78/3.84	–
1,5-α-Ara*f*	108.6	82.5	77.9	85.0	67.9	–
(B)	5.17	4.06	3.94	4.07	3.86/3.91	–
1,3,5-α-Ara*f*	109.7	82.3	81.8	85.0	68.1	–
(C)	5.19	4.07	4.27	4.15	3.88/3.90	–
T-β-Xyl*p*	102.3	73.5	79.9	70.3	66.1	–
(D)	4.73	3.40	3.57	3.67	3.24/3.91	–
1,4-β-Xyl*p*	102.7	72.9	75.8	80.2	66.1	–
(E)	4.54	3.48	3.65	3.88	3.23/3.88	–
T-β-Gal*p*	105.2	72.5	74.8	70.2	76.5	62.2
(F)	4.62	3.62	3.89	3.99	3.73	3.82/3.89
1,3-β-Gal*p*	104.9	73.0	82.1	70.1	76.3	62.2
(G)	4.68	3.78	3.73	3.91	3.81	3.86/3.96
1,6-β-Gal*p*	104.6	72.6	75.6	70.5	77.1	70.5
(H)	4.45	3.51	3.76	3.95	3.78	3.88/4.01
1,6-β-Gal*p*-3-*O*-Ac	106.5	72.6	72.8	69.5	72.1	67.2
(H’)	4.53	3.54	5.19	4.05	4.38	3.65/3.81
1,3,6-β-Gal*p*	104.6	73.0	83.2	71.0	76.0	70.5
(I)	4.49	3.53	3.83	4.10	3.91	3.72/3.91
1,2,4-β-Gal*p*	104.4	78.6	74.4	81.2	76.6	62.2
(J)	4.47	3.73	3.92	4.19	3.73	3.92/3.98
T-α-Gal*p*A	100.4	69.8	70.5	71.9	72.9	176.2
(K)	5.09	3.73	3.91	4.26	4.63	–
T-α-Hex*p*A	100.8	70.4	67.3	108.3	146.4	170.2
(K’)	5.01	3.74	4.29	5.80	–	–
1,4-α-Gal*p*A	99.1	69.7	70.6	79.5	72.9	176.2
(L)	5.12	3.96	4.13	4.04	4.71	–
1,3,4-α-Gal*p*A	100.2	69.6	78.4	79.2	72.7	176.2
(M)	5.09	3.74	4.07	4.50	4.74	–
1,2,4-α-Gal*p*A	100.2	77.5	69.9	79.5	72.8	176.8
(N)	5.08	4.12	3.93	4.27	4.63	–
1,4-α-Gal*p*A-6-*O*-Me	99.9	68.8	68.7	79.4	72.9	172.4
(O)	5.26	3.79	4.04	4.49	5.14	–
1,2,3,4-α-Gal*p*A	100.3	77.5	78.1	78.4	72.9	175.9
(P)	5.15	4.06	4.08	4.44	4.74	–
1,4-β-Gal*p*A(red)	97.7	72.4	73.4	79.1	75.4	175.6
(Q)	4.52	3.38	3.64	4.38	4.06	–
1,4-α-Gal*p*A(red)	93.6	68.7	69.4	79.3	71.8	175.3
(Q’)	5.22	3.71	3.98	4.44	4.43	–
1,2-β-Glc*p*A	106.2	78.2	76.6	73.3	77.9	175.9
(R)	4.50	3.75	3.53	3.54	3.76	–
T-α-Rha*p*	101.1	71.6	70.3	73.6	69.6	17.6
(S)	4.95	4.11	3.90	3.61	3.71	1.18
1,2-α-Rha*p*	101.1	77.9	70.1	73.6	69.6	17.9
(T)	4.93	4.27	3.93	3.64	3.79	1.22
1,3-α-Rha*p*	101.1	71.0	78.2	72.9	69.9	18.1
(U)	4.91	4.24	3.93	3.56	3.78	1.25
1,2,3,4-α-Rha*p*	101.1	79.1	77.8	77.2	69.6	18.6
(V)	4.93	4.31	3.95	3.74	3.99	1.26
T-α-Fuc*p*-2-*O-*Me	99.1	78.9	69.7	71.9	67.8	16.6
(W)	5.66	3.85	3.77	4.05	4.36	1.22
1,3,4-α-Fuc*p*	100.5	68.3	78.6	82.6	68.9	16.6
(X)	5.01	3.92	4.04	3.85	4.10	1.22
1,2,3-α-Ara*p*	109.1	79.2	82.5	69.9	63.5	–
(Y)	5.08	4.12	4.02	4.08	3.66/3.72	–
1,3-β-Api*f*	109.8	77.6	80.3	73.8	71.2	–
(Z)	5.15	4.06	–	4.02/3.80	3.82/3.68	–

**Fig. 5:**
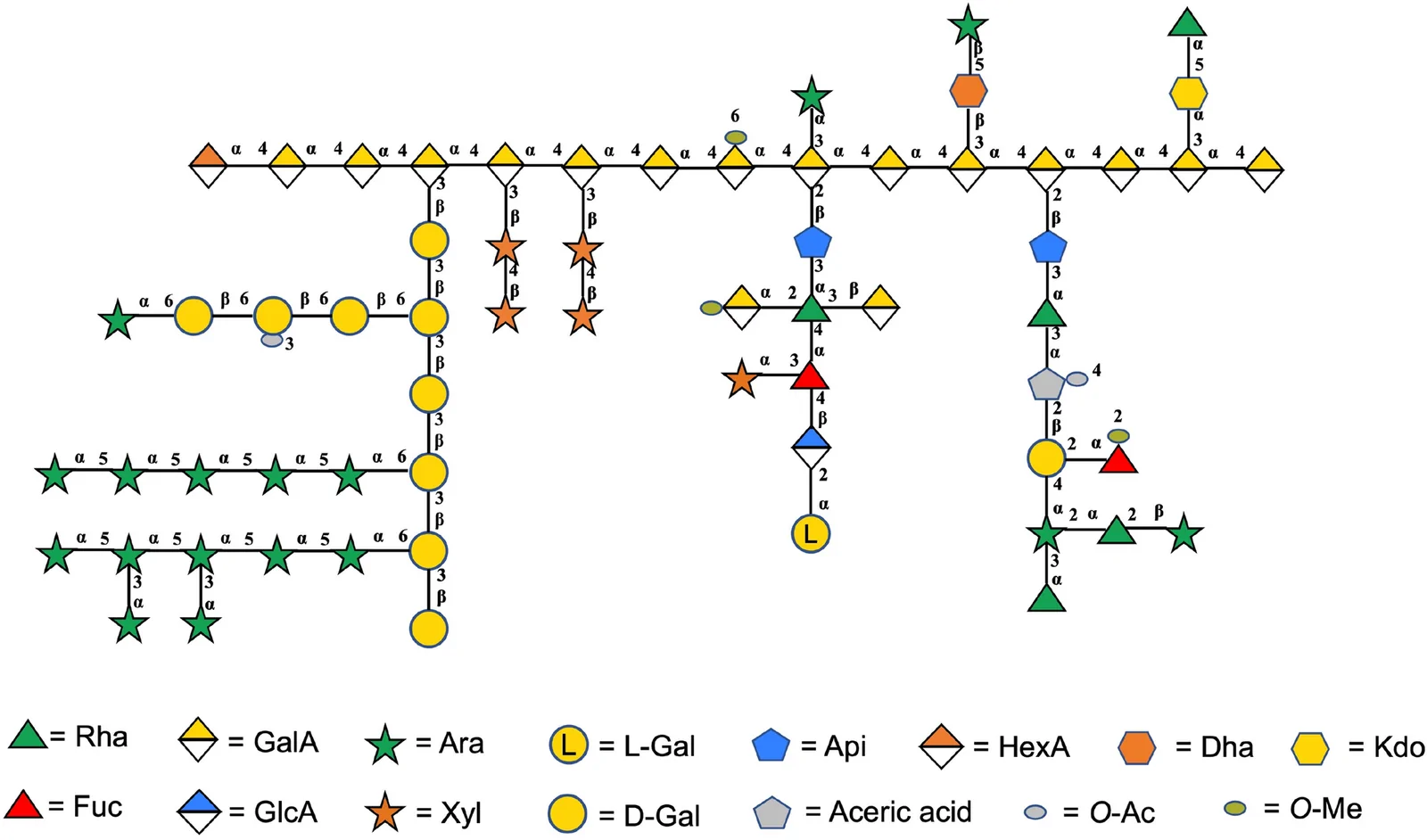
The tentative structural model of PTPS-1-3

To facilitate accurate signal assignment, the HSQC (red) and HMBC (black) spectra of PTPS-1-3 have been overlapped and shown in Fig. 4a, enabling intuitive visualization of correlated cross-peaks [22]. The HSQC spectrum provided one-bond ^1^H–^13^C correlations for individual residues, while the HMBC spectrum revealed long-range correlations that were critical for establishing inter-residue glycosidic linkages. Based on diagnostic HMBC correlations between anomeric protons and linkage carbons, transglycosidic connectivities among adjacent glycosyl residues were assigned, corresponding to linkages numbered 1–32 in Additional file 1: Table S4. These correlations provided direct spectroscopic evidence supporting the linkage patterns inferred from methylation analysis. Collectively, the combined NMR data demonstrates that PTPS-1-3 is a complex pectic polysaccharide containing experimentally established AG-II-rich neutral side-chain regions and HG segments, together with RG-II-related structural features. The assignments of the AG-II and HG domains were directly supported by the present compositional, linkage, and NMR analyses. In contrast, several characteristic signals attributed to the RG-II domain were identified in the NMR spectra of PTPS-1-3 based on comparison with previously reported RG-II and RG-II-containing pectic polysaccharides. Accordingly, on the basis of the experimentally established AG-II and HG structural features, together with the literature-supported assignment of RG-II-related signals, a tentative structural model of PTPS-1-3 is proposed and illustrated in Fig. 5.

### Effects of PTPS-1-3 on BMDC viability, cytokine production, and functional maturation

**Fig. 6:**
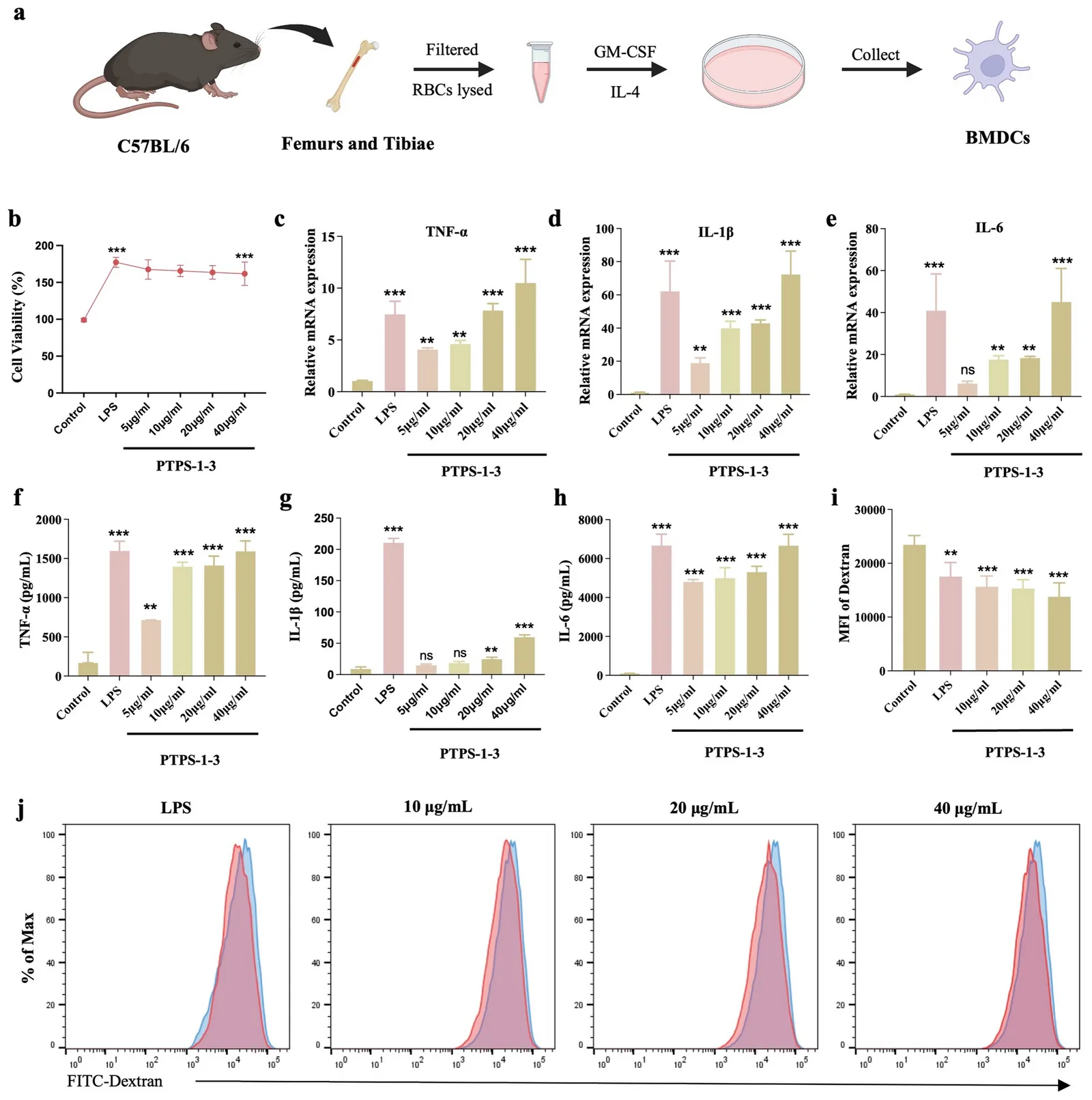
PTPS-1-3 promotes activation and functional maturation of BMDCs. **a** The preparation and differentiation process of BMDCs. **b** Effect of PTPS-1-3 on BMDC viability as determined by the CCK-8 assay. **c–e** Relative mRNA expression levels of pro-inflammatory cytokines TNF-α, IL-1β and IL-6 in BMDCs after stimulation with PTPS-1-3. **f**–**h** Secretion levels of TNF-α, IL-1β and IL-6 in the culture supernatants of BMDCs treated with PTPS-1-3, as determined by ELISA. **i**–**j** Phagocytic activity of BMDCs evaluated by FITC-dextran uptake. Representative flow cytometry histograms (**j**) and quantitative analysis of mean fluorescence intensity (MFI) (**i**) are shown. Data are means ± SD (n = 5). **P < 0.01, ***P < 0.001 compared to Control group

The preparation and differentiation process of BMDCs is schematically illustrated in Fig. 6a. The effect of PTPS-1-3 on BMDC viability was first evaluated using a CCK-8 assay. As shown in Fig. 6b, treatment with PTPS-1-3 at concentrations ranging from 5 to 40 μg/mL for 24 h significantly increased cell viability, reaching approximately 150%–190% of the control level. Notably, cell viability remained above the control level at all tested concentrations, indicating that PTPS-1-3 enhanced BMDC viability without detectable cytotoxicity within this concentration range. Cytokine secretion is a hallmark of DC activation and plays a crucial role in shaping the direction and magnitude of adaptive immune responses [28]. To further assess the immunomodulatory activity of PTPS-1-3, the expression of key pro-inflammatory cytokines in BMDCs was examined. Quantitative real-time PCR analysis showed that the mRNA expression levels of these cytokines were significantly up-regulated following PTPS-1-3 stimulation (Fig. 6c–e). Consistently, ELISA results demonstrated that PTPS-1-3 treatment markedly increased the secretion of TNF-α, IL-1β and IL-6 in the culture supernatants compared with the untreated control group (Fig. 6f–h). These results indicate that PTPS-1-3 effectively stimulates cytokine production in BMDCs. Given that functional maturation of DCs is often accompanied by alterations in antigen uptake, the phagocytic capacity of BMDCs was further evaluated. As shown in Fig. 6i–j, PTPS-1-3 treatment significantly reduced FITC-dextran uptake compared with the control group, indicating a decrease in phagocytic capacity. This reduction is consistent with the transition of immature DCs from an antigen-capturing state toward a mature antigen-presenting phenotype.

**Fig. 7:**
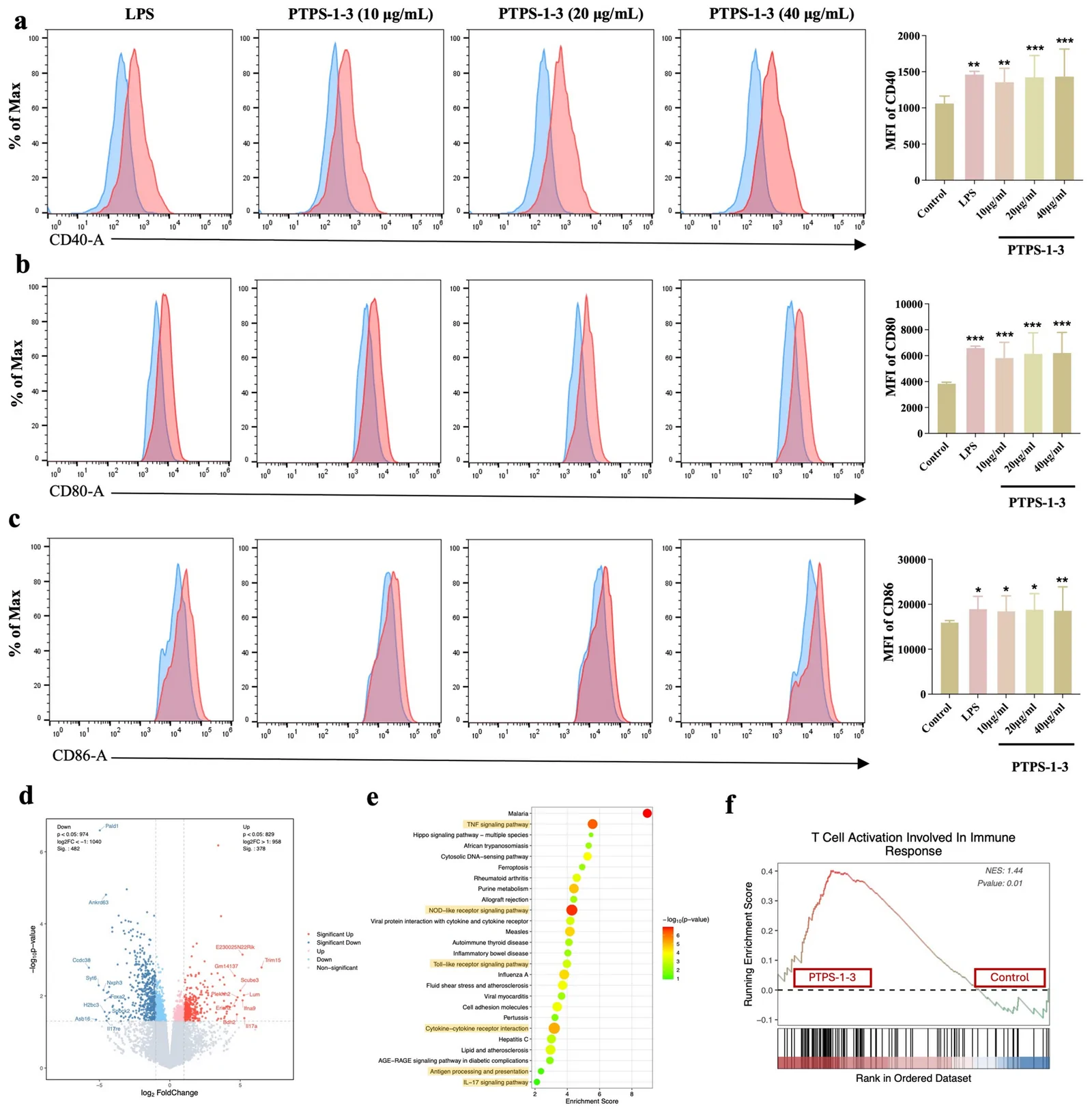
PTPS-1-3 promotes BMDC activation through upregulation of co-stimulatory molecules and immune-related transcriptional reprogramming. Surface expression of CD40 **a**, CD80 **b**, and CD86 **c** on BMDCs following stimulation with PTPS-1-3 at different concentrations (10, 20, and 40 μg/mL) or LPS, as analyzed by flow cytometry. **d** Volcano plot showing differentially expressed genes in BMDCs following PTPS-1-3 treatment compared with the control group. **e** KEGG pathway enrichment analysis of DEGs, revealing the activation of immune-related pathways associated with antigen processing and presentation, T cell activation, and cytokine-mediated signaling. **f** Gene set enrichment analysis (GSEA) demonstrating enrichment of the T cell activation involved in immune response pathway in PTPS-1-3-treated BMDCs. Data are means ± SD (n = 5). **P < 0.01, ***P < 0.001 compared to control group

In parallel, flow cytometric analysis showed that PTPS-1-3 markedly increased the surface expression of the co-stimulatory molecules CD40, CD80 and CD86 on CD11c⁺ BMDCs (Fig. 7a–c). The up-regulation of these maturation-associated markers, together with increased cytokine production and reduced phagocytic activity, demonstrates that PTPS-1-3 promotes both phenotypic and functional maturation of BMDCs.

To further explore the transcriptional changes associated with PTPS-1-3-induced BMDC maturation, RNA-sequencing analysis was performed. Differential expression analysis revealed that PTPS-1-3 treatment induced substantial transcriptional alterations in BMDCs compared with the control group (Fig. 7d). KEGG pathway enrichment analysis indicated that the DEGs were mainly associated with immune-related pathways, including antigen processing and presentation, cytokine–cytokine receptor interaction, cell adhesion molecules, and T cell activation-related pathways (Fig. 7e). Consistently, GSEA revealed significant enrichment of the “T cell activation involved in immune response” gene set in PTPS-1-3-treated BMDCs (Fig. 7f). These transcriptomic changes were consistent with the observed phenotypic maturation of BMDCs and enhanced antigen-specific OT-I CD8⁺ T-cell activation, suggesting that PTPS-1-3 may regulate immune-related transcriptional programs associated with dendritic cell function.

### PTPS-1-3 enhances BMDC-mediated antigen-specific CD8⁺ T cell activation

**Fig. 8:**
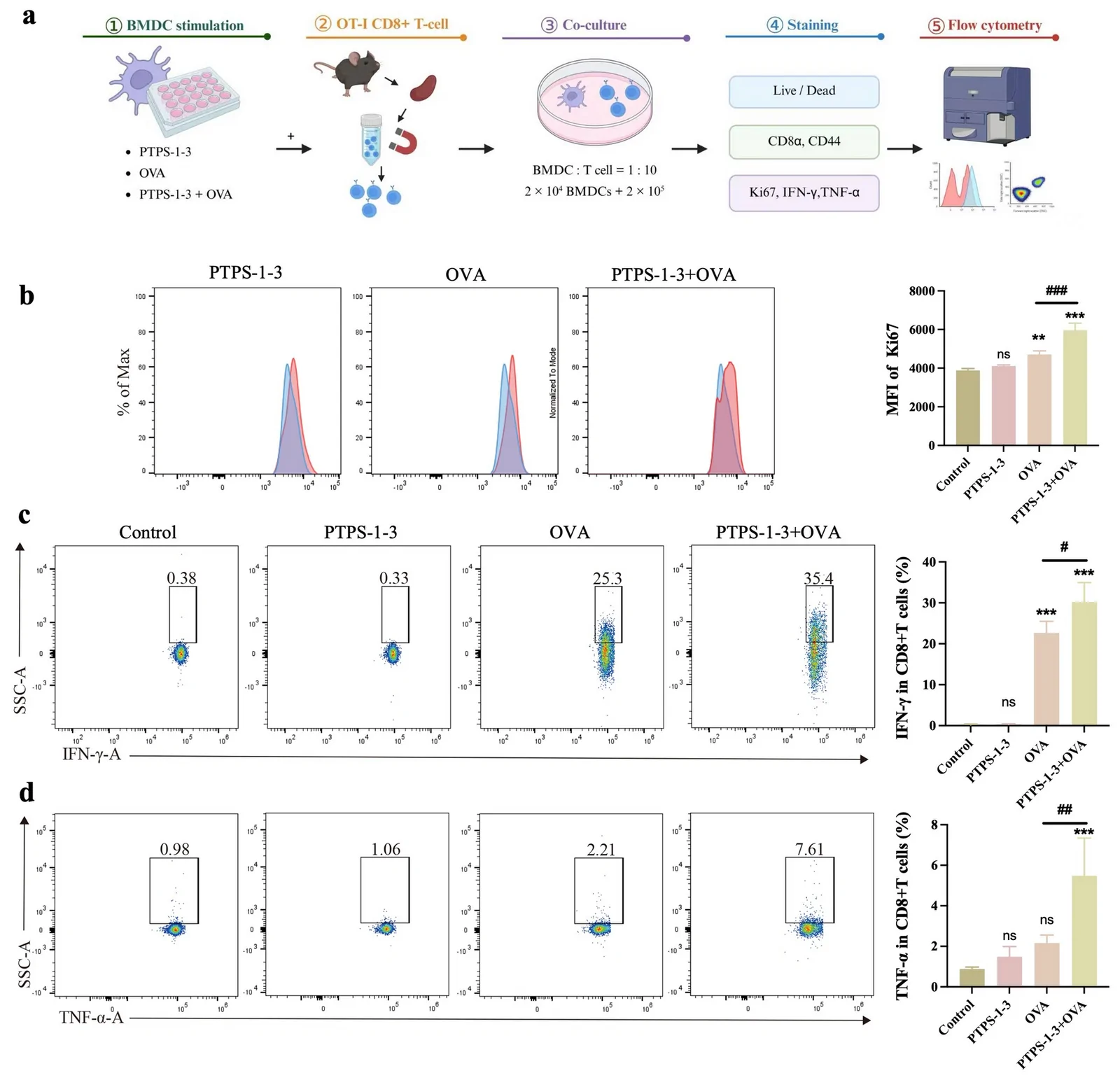
PTPS-1-3 potentiates OVA-induced OT-I CD8⁺ T-cell activation mediated by BMDCs. Experimental design of the co-culture assay in which OT-I CD8⁺ T cells were cultured with BMDCs pretreated with medium, PTPS-1-3, OVA, or PTPS-1-3 plus OVA **a**. Representative flow cytometry plots and quantitative analyses of T cell proliferation and activation, including Ki67⁺ CD8⁺ T cells **b**, IFN-γ⁺ CD8⁺ T cells **c**, and TNF-α⁺ CD8⁺ T cells **d**. Data are presented as means ± SD. ns, not significant; **P < 0.01, ***P < 0.001 compared to control group; #*P* < 0.05, ##* P* < 0.01, and ###* P* < 0.001 compared with the OVA group

To validate whether PTPS-1-3-induced BMDC maturation could enhance antigen-specific T cell activation, an OT-I CD8⁺ T cell co-culture assay was performed. The experimental workflow is shown in Fig. 8a. Briefly, BMDCs were pretreated with medium, PTPS-1-3, ovalbumin (OVA), or PTPS-1-3 plus OVA, followed by co-culture with CD8α⁺ T cells isolated from OT-I mice.

As shown in Fig. 8b, Ki67 staining demonstrated that PTPS-1-3 plus OVA significantly promoted the proliferation of OT-I CD8⁺ T cells compared with OVA alone. In addition, OVA-loaded BMDCs induced IFN-γ production in OT-I CD8⁺ T cells, whereas PTPS-1-3 combined with OVA further increased the proportion of IFN-γ⁺ CD8⁺ T cells (Fig. 8c). A similar pattern was observed for TNF-α production, with the PTPS-1-3 plus OVA group showing a markedly higher percentage of TNF-α⁺ CD8⁺ T cells than the OVA group (Fig. 8d). In contrast, PTPS-1-3 alone did not substantially activate OT-I CD8⁺ T cells in the absence of OVA. These findings indicate that PTPS-1-3 enhances the antigen-presenting capacity of BMDCs and promotes antigen-specific CD8⁺ T cell proliferation and effector cytokine production.

### Structure–activity relationship analysis of PTPS-1-3 in BMDC maturation

**Fig. 9:**
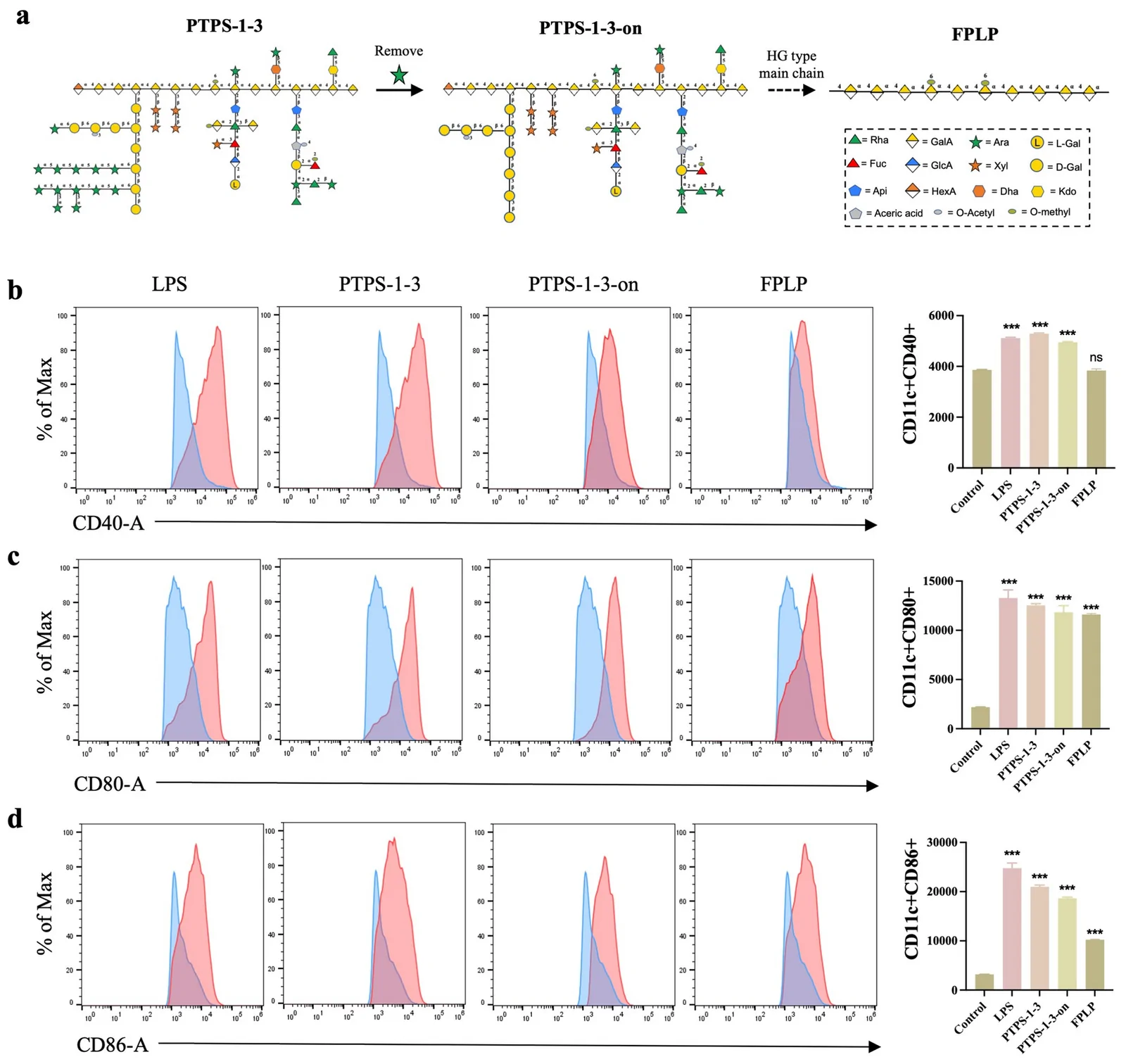
Structure–activity relationship analysis of PTPS-1-3 in BMDC maturation. **a** Proposed structural models of PTPS-1-3 and its partial acid hydrolysate PTPS-1-3-on. Partial acid hydrolysis removed Ara-rich side chains from PTPS-1-3, resulting in an HG-enriched structure. FPLP [29], a previously characterized HG-type polysaccharide from *Ficus pumila* L. fruit with a structure similar to the HG-like regions of PTPS-1-3. (b-d) BMDCs were treated with LPS, FPLP, PTPS-1-3-on or PTPS-1-3 for 24 h, and the surface expression of maturation-associated co-stimulatory molecules was analyzed by flow cytometry. Representative histograms and quantitative analysis showing the expression levels of CD40 **b**, CD80 **c** and CD86 **d** on CD11c⁺ BMDCs. Data are presented as means ± SD. ns, not significant; ***P < 0.001 compared with the control group

To explore the structural features responsible for the BMDC-maturing activity of PTPS-1-3, a preliminary structure–activity relationship analysis was performed by comparing intact PTPS-1-3 with its partial acid hydrolysate PTPS-1-3-on and FPLP, a previously characterized homogalacturonan (HG)-type polysaccharide from *Ficus pumila* L. fruit [29]. PTPS-1-3-on was generated by partial acid hydrolysis of PTPS-1-3 and mainly retained acid-resistant structural segments with reduced arabinose-rich side-chain regions (Fig. 9a). FPLP was included as a structurally related HG-type polysaccharide because its GalA-rich backbone resembles the HG-like regions of PTPS-1-3, but it lacks the complex AG-II-rich side chains and RG-II-related structural features of PTPS-1-3 (Fig. 9b).

BMDCs were treated with LPS, FPLP, PTPS-1-3-on or intact PTPS-1-3, and the expression of co-stimulatory molecules was analyzed by flow cytometry. As shown in Fig. 9c–e, intact PTPS-1-3 induced the strongest up-regulation of CD40, CD80 and CD86 among the tested polysaccharides. FPLP showed relatively weak activity, suggesting that the HG-like backbone alone is insufficient to fully account for the BMDC-maturing effect of PTPS-1-3. PTPS-1-3-on also displayed weaker activity than intact PTPS-1-3, indicating that partial acid hydrolysis and the accompanying loss of labile side-chain regions reduced its immunostimulatory activity. These results suggest that the intact domain organization of PTPS-1-3, especially its AG-II-rich neutral side-chain regions and RG-II-related structural features, contributes substantially to its ability to promote BMDC maturation.

Collectively, these results demonstrate that PTPS-1-3 is a potent BMDC-maturing pectic polysaccharide that enhances cytokine production, up-regulates co-stimulatory molecules, reduces phagocytic activity and promotes antigen-specific CD8⁺ T cell activation. The comparative analysis with PTPS-1-3-on and FPLP suggests that the immunomodulatory activity of PTPS-1-3 may be associated with its intact and highly branched pectic structural features. However, the influence of molecular weight reduction during partial acid hydrolysis cannot be completely excluded, and the observed activity differences may reflect the combined effects of structural alterations and molecular size.

## Discussion

DCs are key antigen-presenting cells that bridge innate and adaptive immunity, and their maturation is essential for initiating effective immune responses [30]. Plant-derived pectic polysaccharides have attracted increasing attention as natural immune-supporting ingredients because of their structural diversity, biocompatibility and immunomodulatory potential [31]. In this study, PTPS-1-3, a structurally complex pectic polysaccharide from *Typha angustifolia* pollen, was characterized and evaluated for its ability to regulate BMDC maturation and antigen-presenting function.

Structural analysis showed that PTPS-1-3 is a low-molecular-weight acidic pectic polysaccharide mainly composed of Rha, Ara, Xyl, Gal and GalA. Methylation analysis and multidimensional NMR spectroscopy indicated that PTPS-1-3 contains AG-II-rich neutral side-chain regions, HG segments and diagnostic RG-II-related structural features. These structural domains may collectively contribute to its immunological activity [32, 33]. AG-II-rich side chains provide highly branched β-galactan and arabinofuranosyl structures that may favor multivalent interactions with pattern-recognition receptors on immune cells. Meanwhile, GalA-containing HG regions and RG-II-related motifs may further contribute to the acidic character, conformational complexity and receptor-interaction capacity of PTPS-1-3.

Functionally, PTPS-1-3 effectively promoted BMDC maturation. It showed no detectable cytotoxicity within the tested concentration range and increased BMDC viability. PTPS-1-3 also significantly enhanced the mRNA expression and secretion of TNF-α, IL-1β and IL-6, indicating activation of pro-inflammatory immune responses. In parallel, PTPS-1-3 up-regulated the expression of the co-stimulatory molecules CD40, CD80 and CD86 on CD11c⁺ BMDCs, while reducing FITC-dextran uptake. These changes are typical features of DC maturation, reflecting a functional transition from antigen capture toward antigen presentation [34]. More importantly, PTPS-1-3-induced BMDC maturation was associated with enhanced antigen-presenting function. Transcriptomic analysis showed that PTPS-1-3 treatment enriched immune-related pathways, including antigen processing and presentation, cytokine-mediated signaling and T cell activation. This was further supported by the OT-I CD8⁺ T cell co-culture assay. Compared with OVA-loaded BMDCs, BMDCs treated with PTPS-1-3 plus OVA induced higher proportions of IFN-γ⁺, TNF-α⁺ and Ki67⁺ CD8⁺ T cells. In contrast, PTPS-1-3 alone did not substantially activate OT-I CD8⁺ T cells without OVA, suggesting that PTPS-1-3 mainly enhances BMDC-mediated antigen presentation rather than directly inducing non-specific T cell activation [35].

The preliminary structure–activity relationship analysis provides evidence that the structural integrity of PTPS-1-3 may contribute to its BMDC-maturing activity. FPLP, a previously characterized HG-type polysaccharide with a GalA-rich backbone similar to the HG-like regions of PTPS-1-3 [29], exhibited weaker activity in inducing CD40, CD80, and CD86 expression, suggesting that an HG-like backbone alone may not be sufficient to achieve the maximal immunomodulatory activity observed for PTPS-1-3. Similarly, PTPS-1-3-on, the partial acid hydrolysate of PTPS-1-3 with reduced acid-labile side-chain regions, displayed lower activity than intact PTPS-1-3. However, partial acid hydrolysis may also result in a reduction in molecular weight, which is known to influence polysaccharide–receptor interactions and immune activity. Therefore, the reduced activity of PTPS-1-3-on may reflect the combined effects of structural alterations and molecular weight reduction. Collectively, these comparisons suggest that the intact pectic architecture, including AG-II-rich side-chain regions and RG-II-related structural features, may contribute to the BMDC-maturing activity of PTPS-1-3, although further studies using polysaccharides with comparable molecular weights are required to clarify the precise structure–activity relationship [36].

Several limitations should also be acknowledged. The precise receptor-binding sites of PTPS-1-3 remain unclear, and the pattern-recognition receptors as well as downstream signaling pathways involved in BMDC activation require further investigation. Future studies using receptor-blocking strategies, pathway inhibitors, receptor-deficient models and in vivo validation will be necessary to clarify the molecular mechanisms underlying its immunomodulatory effects. In summary, PTPS-1-3 is an RG-II-containing pectic polysaccharide with pronounced BMDC-maturing activity. It promotes cytokine production, up-regulates co-stimulatory molecules, reduces phagocytic activity and enhances BMDC-mediated antigen-specific CD8⁺ T cell activation. The intact branched pectic architecture of PTPS-1-3 appears to be essential for its immunomodulatory activity, supporting its structural basis for immune cell modulation.

## Conclusions

Pectic polysaccharides are increasingly recognized as important natural bioactive macromolecules; however, the structure–function relationships of RG-II-containing pectic polysaccharides remain incompletely understood. In this study, a structurally complex pectic polysaccharide, PTPS-1-3, was isolated from *Typha angustifolia* pollen and comprehensively characterized. Integrated structural analyses based on methylation analysis and multidimensional NMR spectroscopy indicated that PTPS-1-3 is an RG-II-containing pectic polysaccharide comprising AG-II-rich neutral side-chain regions, HG segments and diagnostic RG-II-related structural features. Functionally, PTPS-1-3 promoted BMDC activation and maturation, as evidenced by enhanced cytokine production, reduced phagocytic capacity and up-regulated expression of the co-stimulatory molecules CD40, CD80 and CD86. Transcriptomic and OT-I co-culture analyses further demonstrated that PTPS-1-3 enhanced BMDC-mediated antigen presentation and promoted antigen-specific CD8⁺ T cell activation and proliferation. Comparative analysis with PTPS-1-3-on and FPLP suggested that the immunomodulatory activity of PTPS-1-3 may be associated with the integrity and complexity of its branched pectic architecture. However, differences in molecular weight and other physicochemical properties may also contribute to the observed activity, and the specific roles of individual structural features, including AG-II-rich side chains and RG-II-associated motifs, require further clarification. Overall, this study provides structural and functional insights into an RG-II-associated complex pectic polysaccharide and lays a foundation for further elucidating the structure–activity relationships of medicinal pectins.

## Supplementary Information

Additional file 1 (DOCX 3417 KB)

## Data Availability

No datasets were generated or analysed during the current study.

## Abbreviations

BMDCs: Bone marrow-derived dendritic cells
HG: Homogalacturonan
RG-I: Rhamnogalacturonan-I
AG-II: Arabinogalactan-II
RG-II: Rhamnogalacturonan-II
PTPS-1-3: Polysaccharide from *Typha angustifolia* pollen
PTPS-1-3-Re: Products after uronic acid reduction of PTPS-1-3
PTPS-1-3-on: Products after partial acid hydrolysis of PTPS-1-3
Kdo: 3-Deoxy-D-manno-octulosonic acid
Dha: 3-Deoxy-D-lyxo-heptulosaric acid
OVA: Ovalbumin
