# Supplementary material for: Structural characterization of an RG-II-associated complex pectic polysaccharide from Typha angustifolia pollen with dendritic cell-modulating activity
Source: Chin Med. 2026 Sep 28;21:224. doi: 10.1186/s13020-026-01515-9 (PMC13617767; doi:10.1186/s13020-026-01515-9)
Supplement: Supplementary file 1 [file 13020_2026_1515_MOESM1_ESM.docx]

**Supplementary data**

**Methods**

**Materials and reagents**

Chromatography media including DEAE Sepharose Fast Flow and Superdex columns were purchased from GE Healthcare (Uppsala, Sweden). RPMI 1640 medium was purchased from Thermo Fisher Scientific Inc. (Waltham, Massachusetts, USA). Fetal bovine serum, penicillin and streptomycin was purchased from Sigma (St. Louis, USA). GM-CSF and IL-4 was purchased from PeproTech (Cranbury, New Jersey, USA). ELISA kits for mouse TNF-α, IL-6, and IL-1β were purchased from Thermo Fisher Scientific. Antibodies against CD11C PerCP-Cy5.5, MHCII-Super Bright 600, and the viability dye Dye‑eFluor™ 780 were purchased from eBioscience (Minneapolis, MN, USA). CD86-APC, CD80-PE, and CD40-Brilliant Violet 421 antibodies were obtained from Biolegend. Fluorescein isothiocyanate-4 kDa dextran (FD-4) was acquired from Sigma-Aldrich (St. Louis, MO, USA). Ovalbumin (OVA), mitomycin C and GolgiStop™ were used for the antigen presentation assay. CD8α (Ly-2) MicroBeads for mouse CD8α⁺ T cell isolation was purchased from Miltenyi Biotec (130-117-044). Mitomycin C was obtained from MedChemExpress (HY-13316), and GolgiStop™ was purchased from BD Biosciences (554724). The eFluor™ 780 viability dye, anti-CD8α-eFluor™ 506 and anti-CD44-PE antibodies were obtained from Thermo Fisher Scientific/eBioscience. Anti-IFN-γ-APC antibody was purchased from BioLegend, and anti-TNF-α-BV421 and anti-Ki67-FITC antibodies were obtained from Thermo Fisher Scientific/eBioscience and BD Pharmingen™, respectively. OT-I mice were used as the source of OVA-specific CD8⁺ T cells. All other reagents were of analytical grade, and ultrapure water was used for chemical analyses, while distilled water was used for biological assays.

**Partial acid hydrolysis**

PTPS-1-3 (100 mg) was dissolved in 0.1 M TFA (5 mL) and hydrolyzed at 100 ^o^C for 60 min. After neutralization with 5 mL of 0.1 M NaOH, the sample was dialyzed with a 1000 Da dialysis bag for 48h. PTPS-1-3-on was obtained after freeze-drying inside the dialysis bag.

**NMR analysis**

| **Table S1. Pulse Sequences and Key Acquisition and Processing Parameters for NMR** | | | | | | | |
| --- | --- | --- | --- | --- | --- | --- | --- |
| NMR | NS | Pulprog | SW (ppm) | O1P | TD | SI | LB (Hz) |
| ^1^H | 16 | zg30 | 20.026 | 6.175 | 64k | 64k | 0.3 |
| ^13^C | 12k | zgpg30 | 238.9 | 105 | 64k | 64k | 1.0 |
| DEPT135 | 3k | deptsp135 | 238.9 | 105 | 64k | 64k | 1.0 |
| HSQC | 16 | hsqcetgpsisp2.2 | 5.9935/238 | 3.3/105 | 2k/256 | 1024/1024 | 0.3/1.0 |
| HMBC | 50 | hmbcpndgf | 5.9935/238 | 3.3/105 | 2k/256 | 1024/1024 | 0.3/1.0 |
| COSY | 6 | cosygpmfgf | 6.0/6.0 | 3.3/3.3 | 2k/256 | 1024/1024 | 1.0/0.3 |
| Note: NS, Number of Scans; Pulprog, Pulse Program; SW, Spectral Width; O1P, Transmitter Frequency Offset; TD, Time Domain points; SI, Size of Frequency Domain; LB, Line Broadening | | | | | | | |

**Results**

**Fig. S1.** Chromatographic purification profiles of PTPS-1-3. (a) Elution profile of PTPS-1 obtained from ion-exchange chromatography [1]. (b) Elution profile of PTPS-1-3 obtained from size-exclusion gel chromatography [2].

**Fig. S2.** Monosaccharide composition analysis of PTPS-1-3, PTPS-1-3-Re and PTPS-1-3-on [2].

**Table S2.** GC-MS analysis of PTPS-1-3, PTPS-1-3-Re, PTPS-1-3-on.

| Polysaccharide | Molar ratio (%) | | | | |
| --- | --- | --- | --- | --- | --- |
|  | Rha | Ara | Xyl | Glc | Gal |
| PTPS- 1-3 | 9.7 | 39.7 | 14.2 | - | 36.4 |
| PTPS- 1-3-Re | 5.9 | 26.9 | 8.6 | 3.5 | 55.1 |
| PTPS- 1-3-on | 11.5 | - | 11.6 | - | 76.9 |

**Table S3.** Glycosidic linkage composition of PTPS-1-3, PTPS-1-3-Re and PTPS-1-3-on.

| Methylated sugers | Linkages | Molar ratio（%） | | |
| --- | --- | --- | --- | --- |
|  |  | PTPS-1-3 | PTPS-1-3-Re | PTPS-1-3-on |
| 2,3,5-Me_3_-Ara*f* | T-Ara*f* | 14.1 | 14.6 | 5.4 |
| 2,3,4-Me_3_- Xyl*p* | T-Xyl*p* | 8.3 | 5.8 | 9.0 |
| 2,3,4-Me_3_-Rha*p* | T-Rha*p* | 2.2 | 3.9 | 3.1 |
| 2,3,4-Me_3_-Fuc*p* | T-Fuc*p* | 2.1 | 1.9 | 3.1 |
| 2,3-Me_2_-Ara*f* | 1,5-Ara*f* | 16.8 | 9.7 | 4.1 |
| 2,3-Me_2_- Xyl*p* | 1,4-Xyl*p* | 8.2 | 3.9 | 9.5 |
| 2,4-Me_2_-Rha*p* | 1,3-Rha*p* | 2.6 | 1.9 | 3.1 |
| 2-Me_1_-Ara*f* | 1,3,5-Ara*f* | 9.9 | 4.2 | - |
| 2,3,4,6-Me_4_-Glc*p* | T-Glc*p* | - | 2.3 | - |
| 2,3-Me_2_-Rha*p* | 1,4-Rha*p* | - | - | 4.5 |
| 2,3,4,6-Me_4_-Gal*p* | T-Gal*p* | 6.5 | 6.6 | 16.6 |
| 2-Me_1_-Rha*p* | 1,3,4-Rha*p* | 4.1 | 2.1 | - |
| 2-Me_1_-Fuc*p* | 1,3,4-Fuc*p* | 3.7 | 1.9 | 4.4 |
| 2,3,6-Me_3_-Gal*p* | 1,4-Gal*p* | - | 10.7 | - |
| 2,4,6-Me_3_-Gal*p* | 1,3-Gal*p* | 5.2 | 4.2 | 9.7 |
| 2,3,4-Me_3_-Gal*p* | 1,6-Gal*p* | 6.5 | 6.2 | 11.5 |
| 2,6-Me_2_-Gal*p* | 1,3,4-Gal*p* | - | 10.6 | - |
| 3,6-Me_2_-Gal*p* | 1,2,4-Gal*p* | 2.1 | 3.7 | - |
| 2,4-Me_2_-Gal*p* | 1,3,6-Gal*p* | 7.7 | 5.8 | 16.1 |

**Fig. S3. PTPS-1-3 methylation Mass analysis.** (**a**) Mass spectra of PMAAs for T-Ara*f*. (**b**) Mass spectra of PMAAs for T-Xyl*p*. (**c**) Mass spectra of PMAAs for T-Rha*p*. (**d**) Mass spectra of PMAAs for T-Fuc*p*. (**e**) Mass spectra of PMAAs for 1,5-Ara*f*. (**f**) Mass spectra of PMAAs for 1,4-Xyl*p*. (**g**) Mass spectra of PMAAs for 1,3-Rha*p*. (**h**) Mass spectra of PMAAs for 1,3,5-Ara*f*. (**i**) Mass spectra of PMAAs for 1,3,4-Rha*p*. (**j**) Mass spectra of PMAAs for T-Glc*p*. (**k**) Mass spectra of PMAAs for T-Gal*p*. (**l**) Mass spectra of PMAAs for 1,3,4-Fuc*p*. (**m**) Mass spectra of PMAAs for 1,4-Gal*p*. (**n**) Mass spectra of PMAAs for 1,3-Gal*p*. (**o**) Mass spectra of PMAAs for 1,6-Gal*p*. (**p**) Mass spectra of PMAAs for 1,3,4-Gal*p*. (**q**) Mass spectra of PMAAs for 1,2,4-Gal*p*. (**r**) Mass spectra of PMAAs for 1,3,6-Gal*p*.

**Fig. S4.** (a) Two-dimensional NMR spectra of PTPS-1-3-on. Overlay of the HSQC (red) and HMBC (black) with only some of the densities labelled to avoid crowding. (b) ^1^H-^1^H COSY spectrum of PTPS-1-3-on. (D. T-β-Xylp; E. 1,4-β-Xylp; F.T-β-Gal*p*; G. 1,3-β-Gal*p*; H. 1,6-β-Gal*p*; H’. 1,6-β-Gal*p*-3-Ac; I. 1,3,6-β-Gal*p*; K. T-α-Gal*p*A; L. 1,4-α-Gal*p*A; M. 1,3,4-α-Gal*p*A; N. 1,2,4-α-Gal*p*A; O. 1,4-α-Gal*p*A-6-OMe; Q. 1,4-β-Gal*p*A(red); Q’. 1,4-α-Gal*p*A(red); R. 1,2-β-Glc*p*A; U. 1,3-α-Rha*p*; W. T-α-Fuc*p*-2-OMe; X. 1,3,4-α-Fuc*p*; Z. 1,3-β-Api*f*)

**Table S4**. Correlation attribution of PTPS-1-3 NMR signals.

| No. | The cross peak (ppm) | Correlation of nuclear magnetic signals of PTPS-1-3 |
| --- | --- | --- |
| 1 | δ 5.12/79.5 | H-1 of 1,4-α-Gal*p*A (L) and C-4 of 1,4-α-Gal*p*A (L) |
| 2 | δ 5.12/79.2 | H-1 of 1,4-α-Gal*p*A (L) and C-4 of 1,3,4-α-Gal*p*A (M) |
| 3 | δ 5.09/79.2 | H-1 of 1,3,4-α-Gal*p*A (M) and C-4 of 1,3,4-α-Gal*p*A (M) |
| 4 | δ 5.12/79.4 | H-1 of 1,4-α-Gal*p*A (L) and C-4 of 1,4-α-Gal*p*A-6-*O*-Me (O) |
| 5 | δ 5.26/78.4 | H-1 of 1,4-α-Gal*p*A-6-*O*-Me (O) and C-4 of 1,2,3,4-α-Gal*p*A (P) |
| 6 | δ 5.15/79.5 | H-1 of 1,2,3,4-α-Gal*p*A (P) and C-4 of 1,4-α-Gal*p*A (L) |
| 7 | δ 5.09/79.5 | H-1 of 1,3,4-α-Gal*p*A (M) and C-4 of 1,2,4-α-Gal*p*A (N) |
| 8 | δ 5.08/79.5 | H-1 of 1,2,4-α-Gal*p*A (N) and C-4 of 1,4-α-Gal*p*A (L) |
| 9 | δ 5.09/79.1 | H-1 of 1,3,4-α-Gal*p*A (M) and C-4 of 1,4-β/α-Gal*p*A(red) |
| 10 | δ 4.68/78.4 | H-1 of 1,3-β-Gal*p* (G) and C-3 of 1,3,4-α-Gal*p*A (M) |
| 11 | δ 4.49/82.1 | H-1 of 1,3,6-β-Gal*p* (I) and C-3 of 1,3-β-Gal*p* (G) |
| 12 | δ 4.68/83.2 | H-1 of 1,3-β-Gal*p* (G) and C-3 of 1,3,6-β-Gal*p* (I) |
| 13 | δ 4.62/83.2 | H-1 of T-β-Gal*p* (F) and C-3 of 1,3,6-β-Gal*p* (I) |
| 14 | δ 4.59/77.4 | H-1 of 1,6-β-Gal*p* (H) and C-6 of 1,3,6-β-Gal*p* (I) |
| 15 | δ 4.45/70.5 | H-1 of 1,6-β-Gal*p*-3-*O*-Ac (H’) and C-6 of 1,6-β-Gal*p* (H) |
| 16 | δ 4.53/70.5 | H-1 of T-α-Ara*f* (A) and C-6 of 1,6-β-Gal*p* (H) |
| 17 | δ 5.24/70.5 | H-1 of 1,5-α-Ara*f* (B) and C-6 of 1,3,6-β-Gal*p* (I) |
| 18 | δ 5.17/70.5 | H-1 of 1,5-α-Ara*f* (B) and C-5 of 1,5-α-Ara*f* (B) |
| 19 | δ 5.17/67.0 | H-1 of T-α-Ara*f* (A) and C-5 of 1,5-α-Ara*f* (B) |
| 20 | δ 5.24/67.0 | H-1 of 1,3,5-α-Ara*f* (C) and C-5 of 1,5-α-Ara*f* (B) |
| 21 | δ 5.19/67.0 | H-1 of 1,3,5-α-Ara*f* (C) and C-5 of 1,3,5-α-Ara*f* (C) |
| 22 | δ 5.24/81.8 | H-1 of T-α-Ara*f* (A) and C-3 of 1,3,5-α-Ara*f* (C) |
| 23 | δ 4.54/78.4 | H-1 of 1,4-β-Xyl*p* (E) and C-3 of 1,3,4-α-Gal*p*A (M) |
| 24 | δ 4.73/80.2 | H-1 of T-β-Xyl*p* (D) and C-3 of 1,4-β-Xyl*p* (E) |
| 25 | δ 4.62/78.2 | H-1 of T-β-Gal*p* (F) and C-2 of 1,2-β-Glc*p*A (R) |
| 26 | δ4.50/82.6 | H-1 of 1,2-β-Glc*p*A (R) and C-4 of 1,3,4-α-Fuc*p* (X) |
| 27 | δ4.73/78.6 | H-1 of T-β-Xyl*p* (D) and C-3 of 1,3,4-α-Fuc*p* (X) |
| 28 | δ5.01/77.2 | H-1 of 1,3,4-α-Fuc*p* (X) and C-4 of 1,2,3,4-α-Rha*p* (V) |
| 29 | δ5.09/79.1 | H-1 of T-α-Gal*p*A (K) and C-2 of 1,2,3,4-α-Rha*p* (V) |
| 30 | δ5.09/77.8 | H-1 of T-α-Gal*p*A (K) and C-3 of 1,2,3,4-α-Rha*p* (V) |
| 31 | δ4.93/80.3 | H-1 of 1,2,3,4-α-Rha*p* (V) and C-3 of 1,3-β-Api*f* (Z) |
| 32 | δ5.15/77.5 | H-1 of 1,3-β-Api*f* (Z) and C-2 of 1,2,3,4-α-Gal*p*A (P) |

**Table S5. qPCR primers used in this study**

| **qRT-PCR primers** | **Sequence (5' →3’)** |
| --- | --- |
| Mouse β-actin Forward | GGCTGTATTCCCCTCCATCG |
| Mouse β-actin Reverse | CCAGTTGGTAACAATGCCATGT |
| Mouse IL-1β Forward | CTGGTACATCAGCACCTCAC |
| Mouse IL-1β Reverse | AGAAACAGTCCAGCCCATAC |
| Mouse TNF-α Forward | AGTGACAAGCCTGTAGCCC |
| Mouse TNF-α Reverse | GAGGTTGACTTTCTCCTGGTAT |
| Mouse IL-6 Forward | TGTATGAACAACGATGATGCACTT |
| Mouse IL-6 Reverse | ACTCTGGCTTTGTCTTTCTTGTTATCT |

**References**

1. Xu Y, Liu Y, Gao W, Liu H, Yang M, Zhu L, et al. Structural characterization of acetylated mannan from Typha angustifolia pollen and the impact of acetylation on its growth-promoting effect toward Lactobacillus johnsonii [J]. *Carbohyd Polym,* **2026**, 385: 125408.
2. Liu Y, Xu Y, Chen W, Gong H, Zhu L, Zhang T, et al. A new method for the quantification of polysaccharide structural domain: Rapid quantification of Rhamnogalacturonan-II by colorimetry [J]. *Carbohyd Polym,* **2025**, 356: 123423.
